# Supplementary material for: Quantifying the causal impact of biological risk factors on healthcare costs
Source: Nat Commun. 2023 Sep 13;14:5672. doi: 10.1038/s41467-023-41394-4 (PMC10499912; doi:10.1038/s41467-023-41394-4)
Supplement: Supplementary file 1 — Supplementary Information File [file 41467_2023_41394_MOESM1_ESM.pdf]

Supplementary Information

Supplementary Figures and Legends

Supplementary Figure 1. Manhattan plot from GWAS of log-transformed annual total healthcare costs in FinnGen.

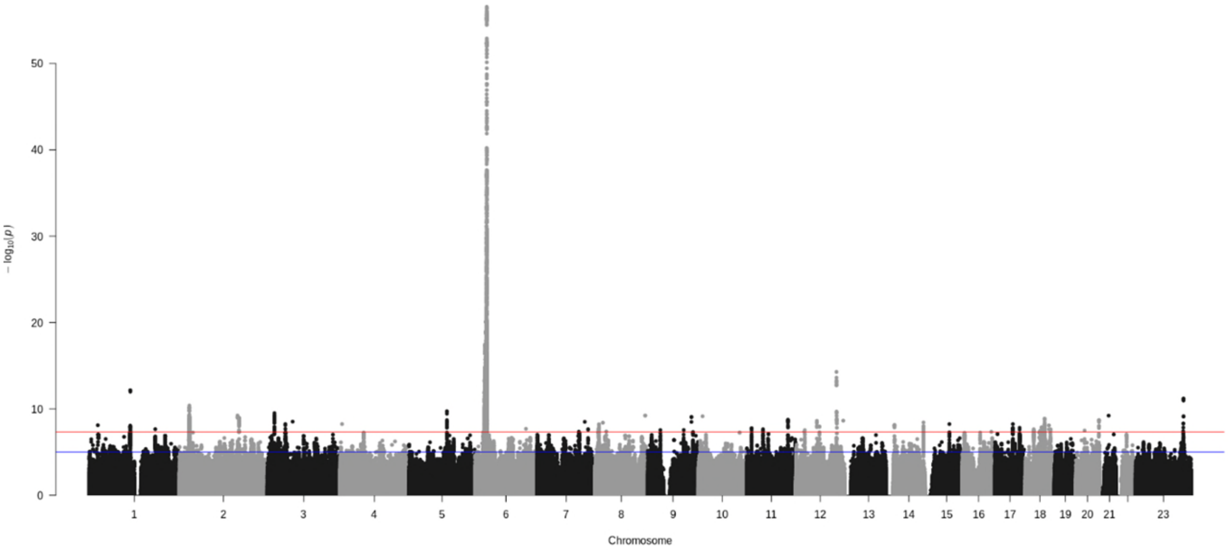

Supplementary Figure 2. Q-Q plot from GWAS of log-transformed annual total healthcare costs in FinnGen. Lambda 0.7 = 1.304, lambda 0.5 = 1.293, lambda 0.1 = 1.285, lambda 0.01 = 1.29, lambda 0.001 = 1.295.

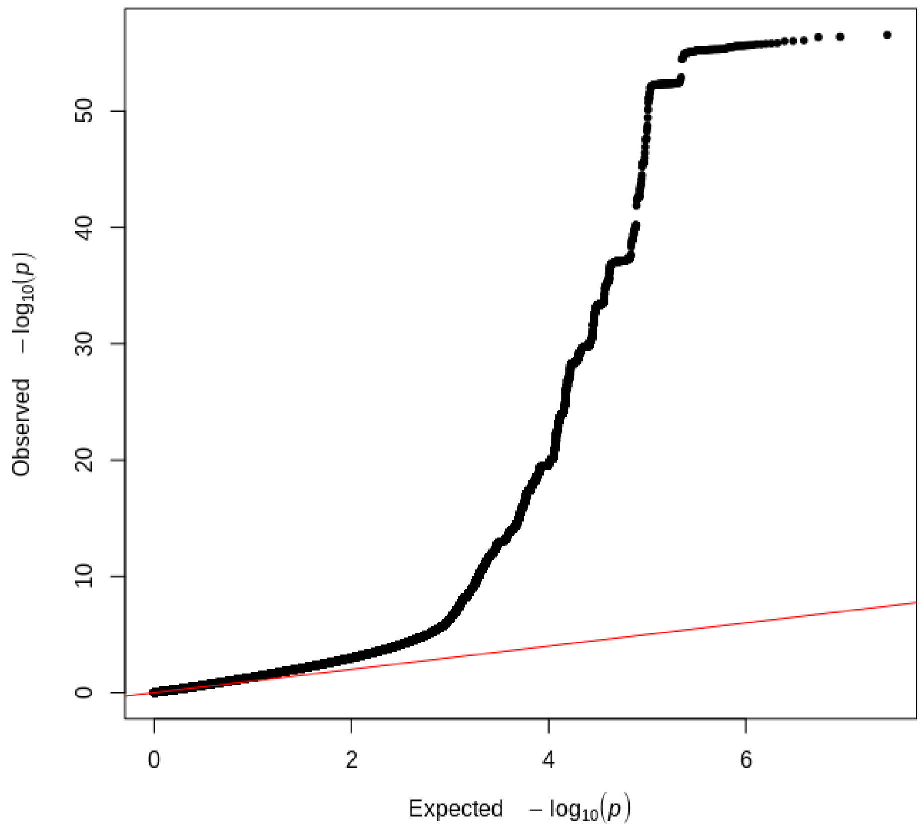

Supplementary Figure 3. Monetary impact of waist circumference for 343,160 FinnGen participants as estimated from Mendelian Randomization at varying baseline healthcare costs. SD is standard deviation. Red point indicates the healthcare costs calculated at the 50th percentile of waist circumference (WC).

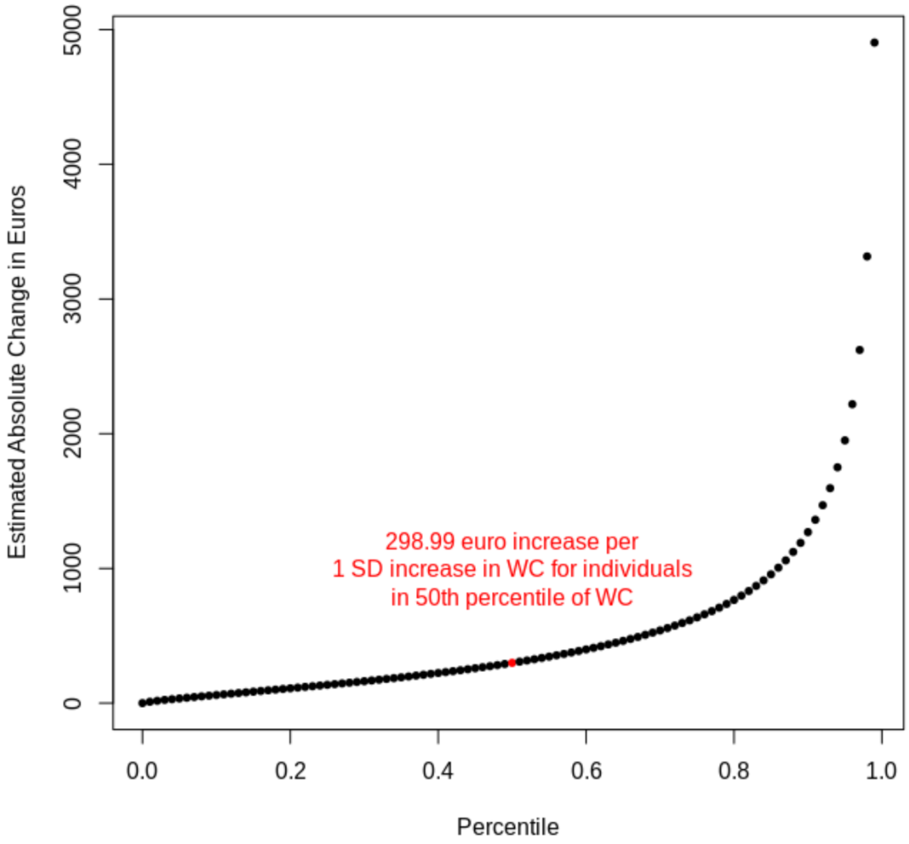

Supplementary Figure 4. Polygenic score analysis between Finland, United Kingdom, and Netherlands for primary, secondary, medication, and total healthcare costs for total costs (A), total costs per year (B), and log total costs per year (C). Points indicate beta effect size estimates and bars indicate 95% confidence interval. Two-sided p-values were calculated from the effect estimates and standard errors of the Mendelian Randomization model and evaluated at alpha = 0.05 without adjustment for multiple testing hypothesis. UK = United Kingdom and NL = Netherlands.

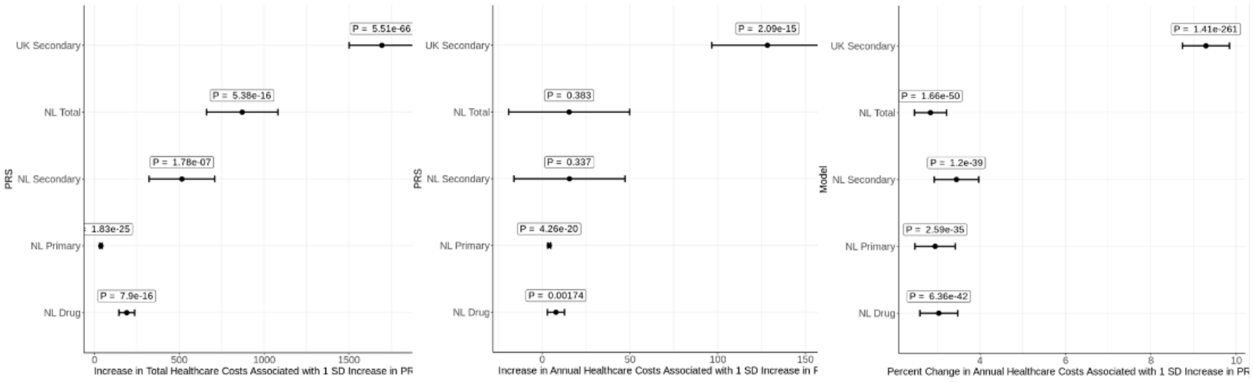

Supplementary Figure 5. Correlation between GWAS- and weighted linear regression-based models in reweighted FinnGen cohort.

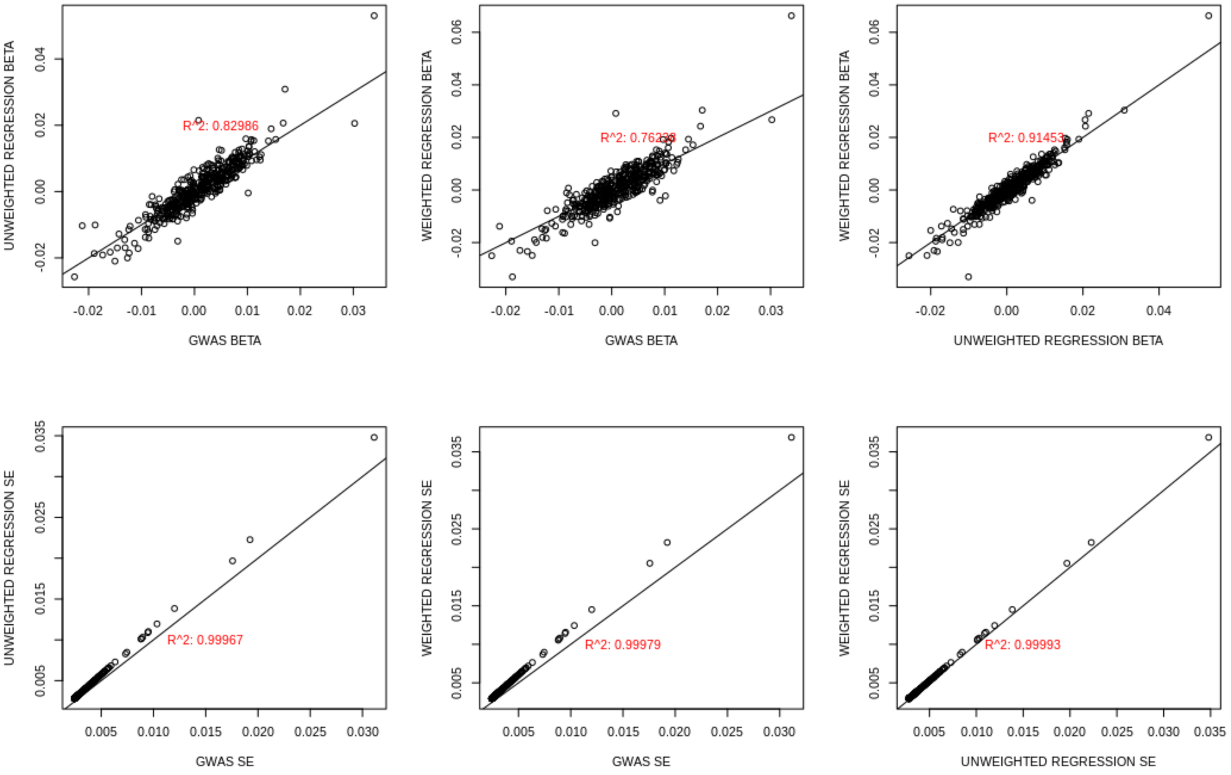

Supplementary Tables

Supplementary Table 1. Summary of summary statistics used as genetic instruments for exposures and mediators.

Phenotype	ID from MRC IEU OpenGWAS Database	Year	Sample size	Population	N SNPs available in sumstats	Unit	Transformation coefficient used to transform SD to raw units	Reference	N SNPs used in MR	R-square value	F-statistic
HDL cholesterol	ieu-a-299	2013	187167	Mixed	2447442	sd	1	http://lipidgenetics.org/	492	0.446178656	305.6735297
HDL cholesterol	ieu-a-299	2013	187167	Mixed	2447442	raw	15.51018433	http://lipidgenetics.org/	492	0.446178656	305.6735297
LDL cholesterol	ieu-a-300	2013	173082	Mixed	2437752	sd	1	http://lipidgenetics.org/	396	0.399169935	289.7114074
LDL cholesterol	ieu-a-300	2013	173082	Mixed	2437752	raw	38.6745912	http://lipidgenetics.org/	396	0.399169935	289.7114074
Triglycerides	ieu-a-302	2013	177861	Mixed	2439433	sd	1	http://lipidgenetics.org/	452	0.372324119	232.8201126
Triglycerides	ieu-a-302	2013	177861	Mixed	2439433	raw	90.72424257	http://lipidgenetics.org/	452	0.372324119	232.8201126
Waist circumference	ieu-a-61	2015	232101	European	2565408	sd	1	https://pubmed.ncbi.nlm.nih.gov/25673412/	42	0.01071664	59.85289658
Waist circumference	ieu-a-61	2015	232101	European	2565408	raw	12.52	https://pubmed.ncbi.nlm.nih.gov/25673412/	42	0.01071664	59.85289658
Body mass index	ieu-a-835	2015	322154	European	2554668	sd	1	https://www.nature.com/articles/nature14177	69	0.01429497	67.69495838
Body mass index	ieu-a-835	2015	322154	European	2554668	raw	4.77	https://www.nature.com/articles/nature14177	69	0.01429497	67.69495838
HDL cholesterol	ieu-b-109	2020	403943	European	12321875	sd	1	https://pubmed.ncbi.nlm.nih.gov/32203549/	362	0.135797258	175.1848019
HDL cholesterol	ieu-b-109	2020	403943	European	12321875	raw	0.323918	https://pubmed.ncbi.nlm.nih.gov/32203549/	362	0.135797258	175.1848019
Triglycerides	ieu-b-111	2020	441016	European	12321875	sd	1	https://pubmed.ncbi.nlm.nih.gov/32203549/	313	0.100635527	157.5492278
Triglycerides	ieu-b-111	2020	441016	European	12321875	raw	1.02441	https://pubmed.ncbi.nlm.nih.gov/32203549/	313	0.100635527	157.5492278
Systolic blood pressure	ieu-b-38	2018	757601	European	7088083	sd	1	https://pubmed.ncbi.nlm.nih.gov/30224653/	461	0.045387404	78.08774463
LDL cholesterol	ieu-b-110	2020	440546	European	12321875	sd	1	https://pubmed.ncbi.nlm.nih.gov/32203549/	177	0.069019757	184.4486365
LDL cholesterol	ieu-b-110	2020	440546	European	12321875	raw	0.434527	https://pubmed.ncbi.nlm.nih.gov/32203549/	177	0.069019757	184.4486365
Systolic blood pressure	ieu-b-38	2018	757601	European	7088083	raw	20.7	https://pubmed.ncbi.nlm.nih.gov/30224653/	461	0.045387404	78.08774463
Systolic blood pressure	ukb-a-360	2017	317754	European	10894596	sd	1	http://www.nealelab.is/uk-biobank	167	0.026764275	52.29766649
Systolic blood pressure	ukb-a-360	2017	317754	European	10894596	raw	19.3261	http://www.nealelab.is/uk-biobank	167	0.026764275	52.29766649
Waist circumference	ukb-a-382	2015	224459	European	2566630	sd	1	http://www.nealelab.is/uk-biobank	232	0.055958971	57.28973833
Waist circumference	ukb-a-382	2015	224459	European	2566630	raw	13.4212	http://www.nealelab.is/uk-biobank	232	0.055958971	57.28973833
Albumin	ukb-d-30600_irnt	2018	385205	European	13585334	quantile	1	https://pan.ukbb.broadinstitute.org/	203	0.042273402	83.71272288
Albumin	ukb-d-30600_raw	2018	385205	European	13585334	raw	1	https://pan.ukbb.broadinstitute.org/	201	0.041610832	83.16346129
Alanine aminotransferase	ukb-d-30620_irnt	2018	420449	European	13586000	quantile	1	https://pan.ukbb.broadinstitute.org/	207	0.040748095	86.23880263
Alanine aminotransferase	ukb-d-30620_raw	2018	420449	European	13586000	raw	1	https://pan.ukbb.broadinstitute.org/	125	0.025493342	87.96605344
Creatinine	ukb-d-30700_irnt	2018	420401	European	13585973	quantile	1	https://pan.ukbb.broadinstitute.org/	337	0.067826081	90.69520111
Creatinine	ukb-d-30700_raw	2018	420401	European	13585973	raw	1	https://pan.ukbb.broadinstitute.org/	184	0.029946233	70.50195393
C-reactive protein	ukb-d-30710_irnt	2018	419693	European	13586004	quantile	1	https://pan.ukbb.broadinstitute.org/	210	0.082240904	179.0000753
C-reactive protein	ukb-d-30710_raw	2018	419693	European	13586004	raw	1	https://pan.ukbb.broadinstitute.org/	53	0.017841629	143.831148
Cystatin C	ukb-d-30720_irnt	2018	420588	European	13586047	quantile	1	https://pan.ukbb.broadinstitute.org/	312	0.109139841	165.0261413
Cystatin C	ukb-d-30720_raw	2018	420588	European	13586047	raw	1	https://pan.ukbb.broadinstitute.org/	196	0.062034264	141.8541338
Glucose	ukb-d-30740_irnt	2018	384760	European	13585279	quantile	1	https://pan.ukbb.broadinstitute.org/	108	0.032547278	119.8196455
Glucose	ukb-d-30740_raw	2018	384760	European	13585279	raw	1	https://pan.ukbb.broadinstitute.org/	61	0.012404914	79.21454429
Glycated hemoglobin	ukb-d-30750_irnt	2018	419434	European	13586180	quantile	1	https://pan.ukbb.broadinstitute.org/	320	0.106851885	156.6894856
Glycated hemoglobin	ukb-d-30750_raw	2018	419434	European	13586180	raw	1	https://pan.ukbb.broadinstitute.org/	190	0.046848766	108.4547446
Lipoprotein(a)	ukb-d-30790_irnt	2018	335796	European	13583854	quantile	1	https://pan.ukbb.broadinstitute.org/	22	0.335032837	7689.715379
Lipoprotein(a)	ukb-d-30790_raw	2018	335796	European	13583854	raw	1	https://pan.ukbb.broadinstitute.org/	21	0.507893887	16502.20565
Vitamin D	ukb-d-30890_irnt	2018	401516	European	13585763	quantile	1	https://pan.ukbb.broadinstitute.org/	59	0.036643376	258.8179126
Vitamin D	ukb-d-30890_raw	2018	401516	European	13585763	raw	1	https://pan.ukbb.broadinstitute.org/	65	0.039433759	253.5472881
Type 2 diabetes	ukb-a-306	2017	336473	European	10894596	sd	1	http://www.nealelab.is/uk-biobank	52	0.010261586	67.07682863
Back pain	ukb-a-473	2017	336650	European	10894596	sd	1	http://www.nealelab.is/uk-biobank	9	0.001084516	40.6097743
Chronic ischemic heart disease	ukb-a-534	2017	337199	European	10894596	sd	1	http://www.nealelab.is/uk-biobank	16	0.003125826	66.07982909
Chronic obstructive pulmonary disease	ukb-a-543	2017	337199	European	10894596	sd	1	http://www.nealelab.is/uk-biobank	2	0.000185913	31.35039071
Stroke	ukb-d-C_STROKE	2018	361194	European	12404026	binary	1	http://www.nealelab.is/uk-biobank	1	8.79E-05	31.76203333
Medication for cholesterol, blood pressure, diabetes, or take exogenous hormones: Blood pressure medication	ukb-b-18009	2018	294710	European	9851867	binary	1	http://www.nealelab.is/uk-biobank	96	0.02833243	56.81972

Supplementary Table 2. Top independent genome-wide significant hits from GWAS of healthcare costs in FinnGen performed using REGENIE. REGENIE fit a whole-genome regression model and performed single-variant association testing, which produces effect estimates, standard errors, and p-values of association between variants and outcomes. P-values were considered at genome-wide significant ($P < 5\times10^{-8}$) to adjust for multiple hypothesis testing.

chr	pos	rsid	ref	alt	pval	beta	se	eaf	info
1	24697088	rs144552623	A	G	7.77E-09	0.064115	0.011105	0.012629	0.962229
1	1.14E+08	rs6679677	C	A	6.92E-13	0.024785	0.003451	0.146586	0.999818
1	1.82E+08	rs11806150	A	G	2.19E-08	-0.02097	0.003747	0.121279	0.996973
2	27111756	rs2891550	C	T	4.14E-11	0.016507	0.002502	0.598099	0.990897
2	28211309	rs72818428	A	G	9.77E-10	0.02183	0.003571	0.135733	0.995658
2	1.59E+08	rs62173233	T	A	5.76E-10	-0.02558	0.004129	0.098028	0.98583
2	1.64E+08	rs1529997	C	T	1.06E-09	0.016677	0.002734	0.723271	0.995827
3	18741984	rs4532156	T	C	3.07E-10	0.016226	0.002577	0.657647	0.99458
3	48670914	rs73078367	C	T	5.64E-09	-0.01732	0.002973	0.214	0.998114
5	1.04E+08	rs2403304	T	C	1.90E-10	0.016368	0.00257	0.346732	0.996614
6	28708617	rs9366724	A	T	1.22E-13	0.032242	0.004348	0.086162	0.998889
6	31434633	rs6935045	T	A	4.52E-13	0.024974	0.00345	0.147252	0.997437
6	31521145	rs549105374	A	T	1.50E-17	-0.03054	0.003582	0.134512	0.996997
6	32624232	rs1281947	A	G	4.40E-57	0.054459	0.00342	0.150316	0.999819
6	32789325	rs9276710	T	C	1.38E-29	0.033907	0.003002	0.20858	0.998497
7	1.4E+08	rs4507685	T	A	2.22E-08	-0.01371	0.002451	0.455723	0.994841
8	10924932	rs4841459	A	C	5.93E-09	-0.01424	0.002446	0.45833	0.995976
8	30931118	rs2344118	C	A	4.11E-08	0.014402	0.002625	0.316921	0.995778
9	34424949	rs62559895	T	C	2.81E-08	0.017022	0.003066	0.199716	0.992107
9	98759624	rs192202574	C	T	2.76E-08	0.051645	0.009295	0.017675	0.994859
9	1.24E+08	rs944339	C	T	4.88E-08	0.013363	0.002449	0.482113	0.994298
11	13298291	rs6486116	A	C	1.75E-08	0.015295	0.002714	0.720071	0.999488
11	44706962	rs10838338	C	T	2.24E-08	-0.02581	0.004615	0.076855	0.986068
11	1.13E+08	rs1836798	A	G	1.91E-09	0.014821	0.002468	0.42046	0.997589
12	24275471	rs10842306	A	G	2.87E-08	-0.01362	0.002454	0.5458	0.998222
12	57584957	rs775251	C	T	2.38E-09	0.01531	0.002565	0.649326	0.994065
12	64702618	rs1690264	C	T	1.08E-08	0.014164	0.002477	0.474906	0.973555
12	1.11E+08	rs3184504	T	C	2.45E-14	-0.01893	0.002483	0.591367	0.999925
14	1.03E+08	rs8010932	A	G	3.77E-09	0.016796	0.00285	0.242823	0.997046
15	67165147	rs2289261	G	C	5.65E-09	0.014383	0.002468	0.57319	0.997683
16	80390010	rs12919036	A	G	4.44E-08	-0.01387	0.002534	0.378044	0.986577
17	49222427	rs55978930	A	G	5.61E-09	0.014792	0.002538	0.370926	0.990835
17	67896391	rs61676547	G	C	1.55E-08	0.016152	0.002856	0.24139	0.99716
18	23496800	rs303754	G	T	2.27E-08	0.013676	0.002447	0.502685	0.993587
18	41733420	rs67311948	T	C	3.51E-08	-0.02267	0.004111	0.097921	0.996927
18	53198529	rs62099231	G	A	1.34E-09	0.014859	0.002451	0.459507	0.998297
20	24379863	rs67976007	G	A	3.21E-08	-0.02409	0.004356	0.087054	0.988078
20	63951116	rs2320226	T	C	2.03E-09	-0.01514	0.002525	0.616045	0.989463

Supplementary Table 3. Full Mendelian Randomization results for all risk factors using all methods in Finland. Mendelian Randomization models produced effect estimates, standard errors, and p-values of causal effects of exposures and outcomes. P-values were adjusted for multiple hypothesis testing with the Bonferroni correction. Table was truncated at 100 rows. See Supplementary Tables for full table.

Exposure	Unit	Outcome	Method	N SNPs	Beta association	Standard error	P-value	Percent change	Confidence intervals for percentage change
Waist circumference	Raw unit	AvoHILMO	Inverse variance weighted	213	0.011647989	0.001533748	3.09E-14	1.17	[0.87, 1.48]
Waist circumference	1 SD	AvoHILMO	Inverse variance weighted	213	0.156329994	0.02058474	3.09E-14	16.92	[12.3, 21.73]
Waist circumference	1 SD	AvoHILMO	Inverse variance weighted	39	0.157797295	0.024504841	1.20E-10	17.09	[11.6, 22.85]
Waist circumference	Raw unit	AvoHILMO	Inverse variance weighted	39	0.012603618	0.001957256	1.20E-10	1.27	[0.88, 1.66]
Body mass index	Raw unit	AvoHILMO	Inverse variance weighted	66	0.024336386	0.004070851	2.26E-09	2.46	[1.65, 3.28]
Body mass index	1 SD	AvoHILMO	Inverse variance weighted	66	0.116084562	0.019417959	2.26E-09	12.31	[8.12, 16.67]
Waist circumference	1 SD	AvoHILMO	Weighted median	213	0.150655502	0.025483785	3.38E-09	16.26	[10.6, 22.21]
Waist circumference	Raw unit	AvoHILMO	Weighted median	213	0.011225189	0.001943269	7.63E-09	1.13	[0.74, 1.51]
Systolic blood pressure	Raw unit	AvoHILMO	Inverse variance weighted	426	0.000199395	3.55E-05	2.01E-08	0.02	[0.01, 0.03]
Systolic blood pressure	1 SD	AvoHILMO	Inverse variance weighted	426	0.004127469	0.000735555	2.01E-08	0.41	[0.27, 0.56]
Waist circumference	1 SD	AvoHILMO	Weighted median	39	0.161155417	0.031453443	3.00E-07	17.49	[10.46, 24.96]
Waist circumference	Raw unit	AvoHILMO	Weighted median	39	0.012871838	0.002556223	4.77E-07	1.3	[0.79, 1.8]
Systolic blood pressure	Raw unit	AvoHILMO	Weighted median	146	0.006214433	0.001294732	1.59E-06	0.62	[0.37, 0.88]
Systolic blood pressure	1 SD	AvoHILMO	Weighted median	146	0.120100759	0.025166939	1.82E-06	12.76	[7.33, 18.46]
Body mass index	Raw unit	AvoHILMO	Weighted median	66	0.025093864	0.005364229	2.90E-06	2.54	[1.47, 3.62]
Body mass index	1 SD	AvoHILMO	Weighted median	66	0.119697733	0.025846461	3.64E-06	12.72	[7.15, 18.57]
Systolic blood pressure	1 SD	AvoHILMO	Inverse variance weighted	146	0.097018559	0.020991154	3.80E-06	10.19	[5.75, 14.82]
Systolic blood pressure	Raw unit	AvoHILMO	Inverse variance weighted	146	0.00502008	0.001086156	3.80E-06	0.5	[0.29, 0.72]
Systolic blood pressure	Raw unit	AvoHILMO	Weighted median	426	0.000226627	4.95E-05	4.70E-06	0.02	[0.01, 0.03]
Systolic blood pressure	1 SD	AvoHILMO	Weighted median	426	0.004691178	0.001034961	5.82E-06	0.47	[0.27, 0.67]
Triglycerides	1 SD	AvoHILMO	Inverse variance weighted	420	0.048058292	0.011033777	1.33E-05	4.92	[2.68, 7.22]
Triglycerides	Raw unit	AvoHILMO	Inverse variance weighted	420	0.000529718	0.000121619	1.33E-05	0.05	[0.03, 0.08]
Alanine aminotransferase	1 SD	AvoHILMO	Inverse variance weighted	170	0.068380093	0.016138862	2.27E-05	7.08	[3.74, 10.52]
Alanine aminotransferase	Raw unit	AvoHILMO	Inverse variance weighted	102	0.005437255	0.001382758	8.42E-05	0.55	[0.27, 0.82]
C-reactive protein	Raw unit	AvoHILMO	Inverse variance weighted	39	0.017461588	0.004628387	0.000161481	1.76	[0.84, 2.69]
Glycated hemoglobin	Raw unit	AvoHILMO	Inverse variance weighted	145	0.008960515	0.002495543	0.000329908	0.9	[0.41, 1.39]
Systolic blood pressure	1 SD	AvoHILMO	MR Egger	426	0.006640303	0.001846283	0.000360413	0.67	[0.3, 1.03]
Systolic blood pressure	Raw unit	AvoHILMO	MR Egger	426	0.000320788	8.92E-05	0.000360413	0.03	[0.01, 0.05]
Waist circumference	Raw unit	AvoHILMO	Weighted mode	213	0.012438049	0.003439055	0.000372937	1.25	[0.57, 1.94]
HDL cholesterol	1 SD	AvoHILMO	Inverse variance weighted	445	-0.035585528	0.010071605	0.00041048	-3.5	[-5.38, -1.57]
HDL cholesterol	Raw unit	AvoHILMO	Inverse variance weighted	445	-0.002294333	0.000649354	0.00041048	-0.23	[-0.36, -0.1]
Waist circumference	1 SD	AvoHILMO	Weighted mode	213	0.166933546	0.047948933	0.000605406	18.17	[7.57, 29.81]
Waist circumference	1 SD	AvoHILMO	MR Egger	213	0.22081111	0.063593569	0.000626058	24.71	[10.09, 41.26]
Waist circumference	Raw unit	AvoHILMO	MR Egger	213	0.016452412	0.004738292	0.000626058	1.66	[0.72, 2.61]
Cystatin C	1 SD	AvoHILMO	Inverse variance weighted	269	0.03052467	0.009180438	0.000884285	3.1	[1.26, 4.97]
Waist circumference	Raw unit	AvoHILMO	Weighted mode	39	0.021059733	0.00588448	0.0009631	2.13	[0.96, 3.31]
C-reactive protein	1 SD	AvoHILMO	Inverse variance weighted	179	0.034401483	0.010538926	0.001097619	3.5	[1.38, 5.66]
Waist circumference	Raw unit	AvoHILMO	Simple mode	39	0.021059733	0.00605371	0.001279398	2.13	[0.92, 3.35]
Cystatin C	Raw unit	AvoHILMO	Inverse variance weighted	166	0.21675076	0.068627916	0.001586667	24.2	[8.57, 42.09]
Waist circumference	1 SD	AvoHILMO	Weighted mode	39	0.263667854	0.077663751	0.001619041	30.17	[11.79, 51.57]
Systolic blood pressure	1 SD	AvoHILMO	Weighted mode	146	0.163766191	0.051578686	0.001829953	17.79	[6.47, 30.32]
Body mass index	1 SD	AvoHILMO	MR Egger	66	0.187071412	0.057559055	0.001841054	20.57	[7.71, 34.97]
Body mass index	Raw unit	AvoHILMO	MR Egger	66	0.039218325	0.012066888	0.001841054	4	[1.57, 6.49]
Waist circumference	1 SD	AvoHILMO	Simple mode	39	0.263667854	0.079831788	0.002091859	30.17	[11.32, 52.22]
Alanine aminotransferase	1 SD	AvoHILMO	Weighted median	170	0.060633163	0.019981172	0.002409229	6.25	[2.17, 10.49]
HDL cholesterol	Raw unit	AvoHILMO	Inverse variance weighted	308	-0.093564365	0.030922704	0.002480174	-8.93	[-14.29, -3.24]
HDL cholesterol	1 SD	AvoHILMO	Inverse variance weighted	308	-0.030307182	0.01001642	0.002480174	-2.99	[-4.87, -1.06]

Glycated hemoglobin	1 SD	AvoHILMO	Inverse variance weighted	256	0.030018343	0.010001361	0.002687186	3.05	[1.05, 5.09]
Body mass index	1 SD	AvoHILMO	Weighted mode	66	0.164177212	0.052978758	0.002868895	17.84	[6.22, 30.74]
Systolic blood pressure	Raw unit	AvoHILMO	Weighted mode	146	0.008473835	0.002843404	0.003379167	0.85	[0.29, 1.41]
Body mass index	Raw unit	AvoHILMO	Weighted mode	66	0.034418703	0.011901304	0.005201318	3.5	[1.12, 5.94]
Systolic blood pressure	Raw unit	AvoHILMO	Weighted mode	426	0.000280712	0.000100328	0.00537648	0.03	[0.01, 0.05]
C-reactive protein	Raw unit	AvoHILMO	MR Egger	39	0.020908101	0.007267671	0.006630153	2.11	[0.67, 3.58]
C-reactive protein	1 SD	AvoHILMO	Weighted mode	179	0.026700055	0.009748048	0.006789337	2.71	[0.76, 4.69]
Systolic blood pressure	1 SD	AvoHILMO	Weighted mode	426	0.005810747	0.002143533	0.006983442	0.58	[0.16, 1.01]
Systolic blood pressure	Raw unit	AvoHILMO	Simple mode	146	0.008473835	0.003225941	0.009545988	0.85	[0.22, 1.49]
Triglycerides	Raw unit	AvoHILMO	Inverse variance weighted	274	0.02603929	0.010680411	0.014767064	2.64	[0.51, 4.81]
Triglycerides	1 SD	AvoHILMO	Inverse variance weighted	274	0.026674909	0.01094112	0.014767064	2.7	[0.52, 4.93]
Waist circumference	1 SD	AvoHILMO	MR Egger	39	0.211799582	0.086399014	0.019073604	23.59	[4.34, 46.4]
Waist circumference	Raw unit	AvoHILMO	MR Egger	39	0.0169169	0.00690088	0.019073604	1.71	[0.34, 3.09]
Systolic blood pressure	1 SD	AvoHILMO	Simple mode	146	0.163766191	0.069151801	0.019193436	17.79	[2.86, 34.89]
Triglycerides	Raw unit	AvoHILMO	Simple mode	420	0.00090712	0.000407746	0.026631999	0.09	[0.01, 0.17]
HDL cholesterol	Raw unit	AvoHILMO	Simple mode	445	-0.004500776	0.002041394	0.027983305	-0.45	[-0.85, -0.05]
HDL cholesterol	1 SD	AvoHILMO	Simple mode	445	-0.069807863	0.032886841	0.03433444	-6.74	[-12.56, -0.53]
Cystatin C	Raw unit	AvoHILMO	Weighted mode	166	0.154710816	0.073771966	0.03750487	16.73	[1.02, 34.89]
Triglycerides	1 SD	AvoHILMO	Simple mode	420	0.082297792	0.040471129	0.042632313	8.58	[0.3, 17.54]
Body mass index	Raw unit	AvoHILMO	Simple mode	66	0.025985283	0.012660946	0.044162024	2.63	[0.12, 5.21]
Body mass index	1 SD	AvoHILMO	Simple mode	66	0.1239498	0.060796689	0.045545247	13.2	[0.48, 27.52]
Systolic blood pressure	1 SD	AvoHILMO	Simple mode	426	0.006138355	0.003094908	0.047968989	0.62	[0.01, 1.23]
Triglycerides	1 SD	AvoHILMO	Weighted mode	420	0.027519008	0.014241992	0.054003135	2.79	[-0.04, 5.7]
Glucose	1 SD	AvoHILMO	Weighted median	88	-0.039584478	0.020723638	0.056118744	-3.88	[-7.71, 0.1]
Systolic blood pressure	1 SD	AvoHILMO	MR Egger	146	0.134657275	0.070006482	0.05639087	14.41	[-0.26, 31.24]
Systolic blood pressure	Raw unit	AvoHILMO	MR Egger	146	0.006967638	0.00362238	0.05639087	0.7	[-0.01, 1.42]
Cystatin C	Raw unit	AvoHILMO	MR Egger	166	0.186798752	0.097377382	0.056810655	20.54	[-0.41, 45.89]
Cystatin C	1 SD	AvoHILMO	Weighted mode	269	0.018803357	0.010083979	0.063319807	1.9	[-0.1, 3.93]
Triglycerides	Raw unit	AvoHILMO	Weighted mode	420	0.000303326	0.000164404	0.065742327	0.03	[0, 0.06]
C-reactive protein	Raw unit	AvoHILMO	Weighted median	39	0.012220736	0.006664778	0.066708931	1.23	[-0.08, 2.56]
Systolic blood pressure	Raw unit	AvoHILMO	Simple mode	426	0.000296539	0.000162894	0.069395211	0.03	[0, 0.06]
Alanine aminotransferase	1 SD	AvoHILMO	Weighted mode	170	0.050719316	0.028048354	0.072340531	5.2	[-0.42, 11.15]
Glucose	Raw unit	AvoHILMO	Inverse variance weighted	52	0.046461514	0.026508185	0.079649511	4.76	[-0.55, 10.34]
C-reactive protein	1 SD	AvoHILMO	Weighted median	179	0.026135799	0.015474028	0.091217377	2.65	[-0.42, 5.81]
Cystatin C	Raw unit	AvoHILMO	Weighted median	166	0.125643426	0.074530989	0.091836694	13.39	[-2.02, 31.22]
LDL cholesterol	Raw unit	AvoHILMO	Weighted mode	151	-0.048593807	0.029189058	0.098041022	-4.74	[-10.04, 0.87]
Waist circumference	Raw unit	AvoHILMO	Simple mode	213	0.009188448	0.005572568	0.100655004	0.92	[-0.17, 2.03]
Alanine aminotransferase	1 SD	AvoHILMO	MR Egger	170	0.049404603	0.029931856	0.100694782	5.06	[-0.92, 11.41]
Cystatin C	1 SD	AvoHILMO	Weighted median	269	0.017123649	0.010447336	0.101204	1.73	[-0.33, 3.83]
C-reactive protein	1 SD	AvoHILMO	MR Egger	179	0.024003723	0.014585055	0.101584604	2.43	[-0.46, 5.4]
LDL cholesterol	1 SD	AvoHILMO	Weighted mode	151	-0.021115321	0.013012947	0.106766383	-2.09	[-4.56, 0.44]
Triglycerides	Raw unit	AvoHILMO	Weighted mode	274	0.019876159	0.012436571	0.111154657	2.01	[-0.45, 4.52]
Cystatin C	1 SD	AvoHILMO	MR Egger	269	0.0203131	0.012711973	0.111236788	2.05	[-0.46, 4.63]
Triglycerides	1 SD	AvoHILMO	Weighted mode	274	0.020361336	0.01274873	0.11139432	2.06	[-0.46, 4.64]
Waist circumference	1 SD	AvoHILMO	Simple mode	213	0.123320002	0.077599028	0.11350652	13.12	[-2.84, 31.71]
LDL cholesterol	Raw unit	AvoHILMO	Weighted median	359	-0.000591731	0.00038365	0.122982963	-0.06	[-0.13, 0.02]
LDL cholesterol	1 SD	AvoHILMO	MR Egger	359	-0.025231894	0.016400426	0.124814649	-2.49	[-5.58, 0.69]
LDL cholesterol	Raw unit	AvoHILMO	MR Egger	359	-0.000652415	0.000424062	0.124814649	-0.07	[-0.15, 0.02]
HDL cholesterol	1 SD	AvoHILMO	Weighted mode	308	-0.01997558	0.01312873	0.129159539	-1.98	[-4.47, 0.58]
Alanine aminotransferase	1 SD	AvoHILMO	Simple mode	170	0.071356397	0.046872315	0.129788585	7.4	[-2.03, 17.73]
HDL cholesterol	Raw unit	AvoHILMO	Weighted mode	308	-0.061668634	0.041015775	0.13372925	-5.98	[-13.24, 1.89]
LDL cholesterol	1 SD	AvoHILMO	Weighted median	359	-0.022884947	0.015567028	0.141537119	-2.26	[-5.2, 0.77]

Supplementary Table 4. Mendelian Randomization replication results in the United Kingdom and Netherlands. Mendelian Randomization models produced effect estimates, standard errors, and p-values of causal effects of exposures and outcomes. P-values were adjusted for multiple hypothesis testing with the Bonferroni correction.

**Phenotype**	**Country**	**Effect size**	**Unit for effect size**	**Confidence interval for effect size**	**P-value**	**Monetary effect**	**Unit for monetary effect**
WC	UK	13.62	cm	[11.66, 15.58]	3.13E-42	162.0849697	per 10 cm
WC	UK	182.82	SD	[156.51, 209.13]	3.13E-42	217.5651553	per SD
WC	UK	7.94	cm	[4.99, 10.89]	1.33E-07	94.49006307	per 10 cm
WC	UK	99.37	SD	[62.44, 136.30]	1.33E-07	118.255385	per SD
BMI	UK	18.66	kg/m^2	[11.54, 25.79]	2.87E-07	111.0317744	per 5 kg/m^2
BMI	UK	89.03	SD	[55.03, 123.03]	2.87E-07	105.9502559	per SD
SBP	UK	0.06	mmHg	[0.009, 0.12]	2.27E-02	0.714030703	per 10 mmHg
SBP	UK	1.30	SD	[0.18, 2.42]	2.27E-02	1.547066524	per SD
SBP	UK	0.74	mmHg	[-0.75, 2.24]	3.31E-01	8.806378674	per 10 mmHg
SBP	UK	14.33	SD	[-14.57, 43.22]	3.31E-01	17.0534333	per SD
WC	NL	0.80	cm	[0.03, 1.57]	4.11E-02	129.907936	per 10 cm
WC	NL	11.24	SD	[0.43, 23.21]	4.11E-02	182.5206501	per SD
WC	NL	0.81	cm	[-0.39, 2.02]	1.85E-01	131.5317852	per 10 cm
WC	NL	10.63	SD	[-4.72, 28.45]	1.85E-01	172.61517	per SD
BMI	NL	3.22	kg/m^2	[0.59, 5.91]	1.60E-02	261.4397212	per 5 kg/m^2
BMI	NL	16.31	SD	[2.86, 31.52]	1.60E-02	264.8498045	per SD
SBP	NL	0.03	mmHg	[0.01, 0.05]	7.69E-03	4.8715476	per 10 mmHg
SBP	NL	0.62	SD	[0.16, 1.08]	7.69E-03	10.06786504	per SD
SBP	NL	0.54	mmHg	[-0.10, 1.190	9.75E-02	87.6878568	per 10 mmHg
SBP	NL	11.00	SD	[-1.89, 25.59]	9.75E-02	178.623412	per SD

Supplementary Table 5. Genetic correlation between Finland, United Kingdom, and Netherlands calculated using LDSC. LDSC calculated genetic correlation based on the deviation between chi-square statistics compared to the expected deviation assuming the null hypothesis.

Country	Cost	Total observed scale h2 (standard error)	Lambda GC	Mean chi^2	Intercept (standard error)	Ratio
FI	Total	0.0649 (0.0034)	1.6831	1.7576	1.2246 (0.0182)	0.2965 (0.024)
FI	Primary care	0.0298 (0.0027)	1.3824	1.4025	1.1599 (0.013)	0.3971 (0.0323)
FI	Secondary care	0.0511 (0.0018)	1.4998	1.5337	1.1604 (0.0099)	0.3006 (0.0185)
FI	Medication	0.0706 (0.0036)	1.7491	1.8654	1.2828 (0.0189)	0.3268 (0.0219)
UK	Secondary care	0.0168 (0.0018)	1.0957	1.118	1.0177 (0.007)	0.1499 (0.0592)
NL	Total	-0.004 (0.0346)	1.0195	1.0231	1.0244 (0.0083)	1.0563 (0.3572)
NL	Primary care	0.0451 (0.0368)	0.9868	0.9941	0.9794 (0.0087)	NA (mean chi^2 < 1)
NL	Secondary care	-0.0015 (0.0362)	1.0046	1.0046	1.0051 (0.0083)	1.1073 (1.7785)
NL	Medication	-0.0059 (0.0354)	1.0315	1.0275	1.0294 (0.0088)	1.0686 (0.3192)

Comparison	Genetic correlation	Stanford error	Z-score	P-value
FI-UK secondary care cost	0.804	0.05492	14.64	1.61E-48
FI-NL total cost	NA	NA	NA	NA
FI-NL primary care cost	0.7694	0.3387	2.2717	0.0231
FI-NL secondary care cost	NA	NA	NA	NA
FI-NL medication cost	NA	NA	NA	NA

Supplementary Table 6. Mendelian Randomization results for reweighted FinnGen cohort. Mendelian Randomization models produced effect estimates, standard errors, and p-values of causal effects of exposures and outcomes. P-values were adjusted for multiple hypothesis testing with the Bonferroni correction.

Exposure	Method	N SNPs	Beta association	Standard error	P-value	Percent change	Confidence interval for percent change
Waist circumference	Inverse variance weighted	192	0.204104803	0.024728054	1.53E-16	22.64	[16.84, 28.73]
Waist circumference	Inverse variance weighted	192	0.015207642	0.001842462	1.53E-16	1.53	[1.17, 1.9]
Body mass index	Inverse variance weighted	63	0.14635652	0.02178195	1.83E-11	15.76	[10.92, 20.81]
Body mass index	Inverse variance weighted	63	0.030682709	0.004566447	1.83E-11	3.12	[2.2, 4.04]
Waist circumference	Weighted median	192	0.184488193	0.028344169	7.57E-11	20.26	[13.76, 27.13]
Waist circumference	Weighted median	192	0.013746028	0.002138211	1.29E-10	1.38	[0.96, 1.81]
Waist circumference	Weighted mode	192	0.206982907	0.041381119	1.28E-06	23	[13.41, 33.39]
Body mass index	Weighted median	63	0.117052329	0.025401271	4.06E-06	12.42	[6.96, 18.16]
Waist circumference	Weighted mode	192	0.015422086	0.003272279	4.69E-06	1.55	[0.9, 2.21]
Body mass index	Weighted median	63	0.024539272	0.005363483	4.76E-06	2.48	[1.41, 3.57]
Systolic blood pressure	Weighted median	130	0.006124076	0.001363666	7.09E-06	0.61	[0.35, 0.88]
Systolic blood pressure	Inverse variance weighted	130	0.120259456	0.027410286	1.15E-05	12.78	[6.88, 19]
Systolic blood pressure	Inverse variance weighted	130	0.006222645	0.001418304	1.15E-05	0.62	[0.34, 0.9]
Systolic blood pressure	Weighted median	130	0.118354501	0.027492358	1.67E-05	12.56	[6.66, 18.8]
Waist circumference	Simple mode	192	0.268090753	0.073968057	0.000371312	30.75	[13.1, 51.14]
Waist circumference	Simple mode	192	0.01997517	0.00572225	0.000598077	2.02	[0.88, 3.17]
Body mass index	Weighted mode	63	0.023356732	0.007814773	0.004010823	2.36	[0.81, 3.94]
Body mass index	Weighted mode	63	0.111411612	0.037986043	0.00469967	11.79	[3.77, 20.43]
Systolic blood pressure	MR Egger	130	0.013284226	0.004759922	0.006062174	1.34	[0.4, 2.29]
Systolic blood pressure	MR Egger	130	0.256732277	0.091990722	0.006062174	29.27	[7.94, 54.81]
Body mass index	MR Egger	63	0.031606496	0.013524084	0.022735747	3.21	[0.51, 5.98]
Body mass index	MR Egger	63	0.150762988	0.06450988	0.022735747	16.27	[2.46, 31.94]
Waist circumference	MR Egger	192	0.146678628	0.074351379	0.049971493	15.8	[0.1, 33.96]
Waist circumference	MR Egger	192	0.010928876	0.005539846	0.049971493	1.1	[0.01, 2.2]
Systolic blood pressure	Weighted mode	130	0.109025055	0.055728594	0.052583655	11.52	[-0.02, 24.39]
Systolic blood pressure	Weighted mode	130	0.005641338	0.002964857	0.05930383	0.57	[-0.02, 1.15]
Body mass index	Simple mode	63	0.088251411	0.054838629	0.112632844	9.23	[-1.9, 21.62]
Body mass index	Simple mode	63	0.018501344	0.012057474	0.130011593	1.87	[-0.51, 4.3]
Systolic blood pressure	Simple mode	130	0.022490297	0.076250349	0.768503662	2.27	[-11.92, 18.76]
Systolic blood pressure	Simple mode	130	0.001163727	0.004103274	0.777164468	0.12	[-0.69, 0.92]

Supplementary Table 7. Multivariable Mendelian Randomization results. Mendelian Randomization models produced effect estimates, standard errors, and p-values of causal effects of exposures and outcomes. P-values were adjusted for multiple hypothesis testing with the Bonferroni correction.

Exposure (adjustment)	N SNPs	Beta association	Standard error	P-value	Percent change	Confidence interval for percent change
Systolic blood pressure	146	0.122910265	0.019480779	2.80E-10	13.08	[8.84, 17.48]
Systolic blood pressure (Back pain)	141	0.137259865	0.019481943	1.85E-12	14.71	[10.41, 19.18]
Systolic blood pressure (Back pain)	6	0.402395219	0.165252438	0.014890412	49.54	[8.17, 106.74]
Systolic blood pressure (Chronic ischemic heart disease)	140	0.117040775	0.020656652	1.46E-08	12.42	[7.96, 17.06]
Systolic blood pressure (Chronic ischemic heart disease)	9	0.490443393	0.325946778	0.132408108	63.3	[-13.79, 209.35]
Systolic blood pressure (Type 2 diabetes)	31	0.562597966	0.179663404	0.001739751	75.52	[23.42, 149.61]
Systolic blood pressure (Type 2 diabetes)	134	0.117334121	0.023976784	9.90E-07	12.45	[7.29, 17.86]
Systolic blood pressure (Chronic obstructive pulmonary disease)	142	0.123009287	0.020044428	8.42E-10	13.09	[8.73, 17.62]
Systolic blood pressure (Chronic obstructive pulmonary disease)	0	1.530639761	2.501787549	0.540657887	362.11	[-96.57, 62174.89]
Systolic blood pressure (Stroke)	142	0.090795774	0.020723857	1.18E-05	9.5	[5.15, 14.04]
Systolic blood pressure (Stroke)	1	3.655106515	0.911265608	6.05E-05	3767.16	[548.2, 22971.59]
Systolic blood pressure (Blood pressure medication)	127	0.037374119	0.031720892	0.238709803	3.81	[-2.45, 10.47]
Systolic blood pressure (Blood pressure medication)	68	0.23510217	0.086576174	0.006616523	26.5	[6.76, 49.9]
Systolic blood pressure (All)	27	0.459606406	0.158018571	0.003630981	58.35	[16.17, 115.83]
Systolic blood pressure (All)	120	0.055467051	0.036945512	0.133272706	5.7	[-1.68, 13.64]
Systolic blood pressure (All)	4	0.532909462	0.17123401	0.001857171	70.39	[21.81, 138.34]
Systolic blood pressure (All)	9	-0.033505085	0.355652629	0.924944462	-3.3	[-51.84, 94.17]
Systolic blood pressure (All)	0	2.76283842	2.189933536	0.207089753	1484.48	[-78.33, 115776.28]
Systolic blood pressure (All)	66	0.084097545	0.109622199	0.442987594	8.77	[-12.26, 34.85]
Systolic blood pressure (All)	0	3.087092868	0.986816671	0.001757998	2091.33	[216.75, 15060.12]
Body mass index	66	0.127848707	0.015407011	1.06E-16	13.64	[10.26, 17.12]
Body mass index (Back pain)	65	0.116856782	0.015456526	4.02E-14	12.4	[9.04, 15.85]
Body mass index (Back pain)	6	0.365900745	0.147175881	0.012913493	44.18	[8.05, 92.39]
Body mass index (Chronic ischemic heart disease)	62	0.12234999	0.018034934	1.17E-11	13.01	[9.09, 17.08]
Body mass index (Chronic ischemic heart disease)	10	0.244999483	0.316010902	0.438169457	27.76	[-31.23, 137.35]
Body mass index (Type 2 diabetes)	62	0.096968449	0.025180776	0.000117688	10.18	[4.88, 15.76]
Body mass index (Type 2 diabetes)	30	0.745895457	0.177961409	2.77E-05	110.83	[48.75, 198.83]
Body mass index (Chronic obstructive pulmonary disease)	65	0.126470693	0.015609499	5.40E-16	13.48	[10.06, 17.01]
Body mass index (Chronic obstructive pulmonary disease)	0	0.497060147	2.969990629	0.86708613	64.39	[-99.51, 55359.7]
Body mass index (Stroke)	65	0.128663231	0.015907781	6.06E-16	13.73	[10.24, 17.33]
Body mass index (Stroke)	0	-0.753460968	1.153163733	0.513507502	-52.93	[-95.09, 351.2]
Body mass index (Blood pressure medication)	54	0.096598673	0.020035366	1.43E-06	10.14	[5.9, 14.55]
Body mass index (Blood pressure medication)	60	0.389751186	0.055064039	1.46E-12	47.66	[32.55, 64.49]
Body mass index (All)	51	0.054651821	0.023755587	0.021414921	5.62	[0.81, 10.65]
Body mass index (All)	21	0.721296717	0.17124299	2.53E-05	105.71	[47.06, 187.75]
Body mass index (All)	4	0.703705715	0.201349662	0.000474162	102.12	[36.21, 199.92]
Body mass index (All)	6	-0.518399586	0.419436346	0.216479601	-40.45	[-73.83, 35.49]
Body mass index (All)	0	8.132679532	2.652704695	0.002170837	340290.82	[1779.1, 61660204.62]
Body mass index (All)	55	0.265555042	0.080976154	0.001040197	30.42	[11.28, 52.85]
Body mass index (All)	0	3.089070444	1.188020459	0.009317409	2095.67	[113.95, 22433.55]
Waist circumference	213	0.205264091	0.01703222	1.90E-33	22.78	[18.75, 26.95]
Waist circumference (Back pain)	206	0.169971559	0.019454333	2.39E-18	18.53	[14.09, 23.13]
Waist circumference (Back pain)	4	0.501044255	0.146122812	0.000605976	65.04	[23.94, 119.78]
Waist circumference (Chronic ischemic heart disease)	203	0.197124449	0.0178098	1.79E-28	21.79	[17.61, 26.12]
Waist circumference (Chronic ischemic heart disease)	10	0.505251208	0.330006304	0.125760738	65.74	[-13.2, 216.47]
Waist circumference (Type 2 diabetes)	32	0.691777821	0.155635892	8.80E-06	99.73	[47.22, 170.97]
Waist circumference (Type 2 diabetes)	191	0.164263405	0.02199847	8.20E-14	17.85	[12.88, 23.05]
Waist circumference (Chronic obstructive pulmonary disease)	207	0.20605183	0.018255047	1.51E-29	22.88	[18.56, 27.36]
Waist circumference (Chronic obstructive pulmonary disease)	1	-0.850323672	1.873014039	0.649838319	-57.27	[-98.91, 1579]
Waist circumference (Stroke)	207	0.195786294	0.018089207	2.67E-27	21.63	[17.39, 26.02]
Waist circumference (Stroke)	1	1.208105223	0.806721981	0.134250618	234.71	[-31.14, 1526.93]
Waist circumference (Blood pressure medication)	170	0.163119674	0.018305122	5.05E-19	17.72	[13.57, 22.02]
Waist circumference (Blood pressure medication)	62	0.404518534	0.054841948	1.63E-13	49.86	[34.59, 66.86]
Waist circumference (All)	26	0.555951057	0.157005888	0.000398679	74.36	[28.17, 137.19]
Waist circumference (All)	156	0.082306721	0.025058516	0.001021342	8.58	[3.37, 14.04]
Waist circumference (All)	3	0.605319527	0.162586242	0.000196817	83.18	[33.2, 151.93]
Waist circumference (All)	10	0.102440911	0.344415221	0.766134936	10.79	[-43.6, 117.6]
Waist circumference (All)	0	0.90511267	2.041623798	0.657527204	147.22	[-95.48, 13419.19]
Waist circumference (All)	59	0.304253238	0.069347137	1.15E-05	35.56	[18.33, 55.3]
Waist circumference (All)	0	2.029282307	0.840352109	0.01574385	660.86	[46.55, 3850.28]

Supplementary Table 8. FinnGen Consortium.

Full Name	Affiliation	E-mail	Role 1	Role 2
Aarno Palotie	Institute for Molecular Medicine Finland (FIMM), HiLIFE, University of Helsinki, Helsinki, Finland; Broad Institute of MIT and Harvard; Massachusetts General Hospital	aarno.palotie@helsinki.fi	Steering Committee	Steering Committee
Mark Daly	Institute for Molecular Medicine Finland (FIMM), HiLIFE, University of Helsinki, Helsinki, Finland; Broad Institute of MIT and Harvard; Massachusetts General Hospital	mark.daly@helsinki.fi	Steering Committee	Steering Committee
Bridget Riley-Gills	Abbvie, Chicago, IL, United States	bridget.rileygillis@abbvie.com	Steering Committee	Pharmaceutical companies
Howard Jacob	Abbvie, Chicago, IL, United States	howard.jacob@abbvie.com	Steering Committee	Pharmaceutical companies
Dirk Paul	Astra Zeneca, Cambridge, United Kingdom	dirk.paul@astrazeneca.com	Steering Committee	Pharmaceutical companies
Athena Matakidou	Astra Zeneca, Cambridge, United Kingdom	athena.x.matakidou@gsk.com	Steering Committee	Pharmaceutical companies
Adam Platt	Astra Zeneca, Cambridge, United Kingdom	adam.platt@astrazeneca.com	Steering Committee	Pharmaceutical companies
Heiko Runz	Biogen, Cambridge, MA, United States	heiko.runz@biogen.com	Steering Committee	Pharmaceutical companies
Sally John	Biogen, Cambridge, MA, United States	sally.john@biogen.com	Steering Committee	Pharmaceutical companies
George Okafo	Boehringer Ingelheim, Ingelheim am Rhein, Germany	george.okafo@boehringer-ingelheim.com	Steering Committee	Pharmaceutical companies
Nathan Lawless	Boehringer Ingelheim, Ingelheim am Rhein, Germany	nathan.lawless@boehringer-ingelheim.com	Steering Committee	Pharmaceutical companies
Heli Salminen-Mankonen	Boehringer Ingelheim, Ingelheim am Rhein, Germany	heli.salminen-mankonen@boehringer-ingelheim.com	Steering Committee	Pharmaceutical companies
Robert Plenge	Bristol Myers Squibb, New York, NY, United States	robert.plenge@bms.com	Steering Committee	Pharmaceutical companies
Joseph Maranville	Bristol Myers Squibb, New York, NY, United States	joseph.maranville@bms.com	Steering Committee	Pharmaceutical companies
Mark McCarthy	Genentech, San Francisco, CA, United States	mccarthy.mark@gene.com	Steering Committee	Pharmaceutical companies
Julie Hunkapiller	Genentech, San Francisco, CA, United States	hunkapiller.julie@gene.com	Steering Committee	Pharmaceutical companies
Margaret G. Ehm	GlaxoSmithKline, Collegeville, PA, United States	meg.g.ehm@gsk.com	Steering Committee	Pharmaceutical companies
Kirsi Auro	GlaxoSmithKline, Espoo, Finland	kirsi.m.auro@gsk.com	Steering Committee	Pharmaceutical companies
Simonne Longerich	Merck, Kenilworth, NJ, United States	simonne.longerich@merck.com	Steering Committee	Pharmaceutical companies
Caroline Fox	Merck, Kenilworth, NJ, United States	caroline.fox@merck.com	Steering Committee	Pharmaceutical companies
Anders Mälarstig	Pfizer, New York, NY, United States	anders.malarstig@pfizer.com	Steering Committee	Pharmaceutical companies
Katherine Klinger	Translational Sciences, Sanofi R&D, Framingham, MA, USA	katherine.klinger@sanofi.com	Steering Committee	Pharmaceutical companies
Deepak Raipal	Translational Sciences, Sanofi R&D, Framingham, MA, USA	deepak.rajpal@sanofi.com	Steering Committee	Pharmaceutical companies
Eric Green	Maze Therapeutics, San Francisco, CA, United States	egreen@mazetx.com	Steering Committee	Pharmaceutical companies
Robert Graham	Maze Therapeutics, San Francisco, CA, United States	rgraham@mazetx.com	Steering Committee	Pharmaceutical companies
Robert Yang	Janssen Biotech, Beerse, Belgium	ryang31@its.jnj.com	Steering Committee	Pharmaceutical companies
Chris O´Donnell	Novartis Institutes for BioMedical Research, Cambridge, MA, United States	chris.odonnell@novartis.com	Steering Committee	Pharmaceutical companies
Tomi P. Mäkelä	HiLIFE, University of Helsinki, Finland, Finland	tomi.makela@helsinki.fi	Steering Committee	University of Helsinki & Biobanks
Jaakko Kaprio	Institute for Molecular Medicine Finland (FIMM), HiLIFE, University of Helsinki, Helsinki, Finland	jaakko.kaprio@helsinki.fi	Steering Committee	University of Helsinki & Biobanks
Petri Virolainen	Auria Biobank / University of Turku / Hospital District of Southwest Finland, Turku, Finland	petri.virolainen@tyks.fi	Steering Committee	University of Helsinki & Biobanks
Antti Hakanen	Auria Biobank / University of Turku / Hospital District of Southwest Finland, Turku, Finland	antti.hakanen@tyks.fi	Steering Committee	University of Helsinki & Biobanks
Terhi Kilpi	THL Biobank / Finnish Institute for Health and Welfare (THL), Helsinki, Finland	terhi.kilpi@thl.fi	Steering Committee	University of Helsinki & Biobanks
Markus Perola	THL Biobank / Finnish Institute for Health and Welfare (THL), Helsinki, Finland	markus.perola@thl.fi	Steering Committee	University of Helsinki & Biobanks
Jukka Partanen	Finnish Red Cross Blood Service / Finnish Hematology Registry and Clinical Biobank, Helsinki, Finland	jukka.partanen@veripalvelu.fi	Steering Committee	University of Helsinki & Biobanks
Anne Pitkäranta	Helsinki Biobank / Helsinki University and Hospital District of Helsinki and Uusimaa, Helsinki	anne.pitkaranta@hus.fi	Steering Committee	University of Helsinki & Biobanks
Taneli Raivio	Helsinki Biobank / Helsinki University and Hospital District of Helsinki and Uusimaa, Helsinki	taneli.raivio@hus.fi	Steering Committee	University of Helsinki & Biobanks
Raisa Serpi	Northern Finland Biobank Borealis / University of Oulu / Northern Ostrobothnia Hospital District, Oulu, Finland	raisa.serpi@ppshp.fi	Steering Committee	University of Helsinki & Biobanks
Tarja Laitinen	Finnish Clinical Biobank Tampere / University of Tampere / Pirkanmaa Hospital District, Tampere, Finland	tarja.laitinen@pshp.fi	Steering Committee	University of Helsinki & Biobanks
Veli-Matti Kosma	Biobank of Eastern Finland / University of Eastern Finland / Northern Savo Hospital District, Kuopio, Finland	veli-matti.kosma@uef.fi	Steering Committee	University of Helsinki & Biobanks
Jari Laukkanen	Central Finland Biobank / University of Jyväskylä / Central Finland Health Care District, Jyväskylä, Finland	jari.laukkanen@ksshp.fi	Steering Committee	University of Helsinki & Biobanks
Marco Hautalahti	FINBB - Finnish biobank cooperative	marco.hautalahti@finbb.fi	Steering Committee	University of Helsinki & Biobanks
Outi Tuovila	Business Finland, Helsinki, Finland	outi.tuovila@businessfinland.fi	Steering Committee	Other Experts/ Non-Voting Members
Raimo Pakkanen	Business Finland, Helsinki, Finland	raimo.pakkanen@businessfinland.fi	Steering Committee	Other Experts/ Non-Voting Members
Jeffrey Waring	Abbvie, Chicago, IL, United States	jeff.waring@abbvie.com	Scientific Committee	Pharmaceutical companies

Bridget Riley-Gillis	Abbvie, Chicago, IL, United States	bridget.rileygillis@abbvie.com	Scientific Committee	Pharmaceutical companies
Fedik Rahimov	Abbvie, Chicago, IL, United States	fedik.rahimov@abbvie.com	Scientific Committee	Pharmaceutical companies
Ioanna Tachmazidou	Astra Zeneca, Cambridge, United Kingdom	ioanna.tachmazidou@astrazeneca.com	Scientific Committee	Pharmaceutical companies
Chia-Yen Chen	Biogen, Cambridge, MA, United States	chiayen.chen@biogen.com	Scientific Committee	Pharmaceutical companies
Heiko Runz	Biogen, Cambridge, MA, United States	heiko.runz@biogen.com	Scientific Committee	Pharmaceutical companies
Zhihao Ding	Boehringer Ingelheim, Ingelheim am Rhein, Germany	zhihao.ding@boehringer-ingelheim.com	Scientific Committee	Pharmaceutical companies
Marc Jung	Boehringer Ingelheim, Ingelheim am Rhein, Germany	marc_oliver.jung@boehringer-ingelheim.com	Scientific Committee	Pharmaceutical companies
Shameek Biswas	Bristol Myers Squibb, New York, NY, United States	Shameek.Biswas@bms.com	Scientific Committee	Pharmaceutical companies
Rion Pendergrass	Genentech, San Francisco, CA, United States	penders2@gene.com	Scientific Committee	Pharmaceutical companies
Julie Hunkapiller	Genentech, San Francisco, CA, United States	hunkapiller.julie@gene.com	Scientific Committee	Pharmaceutical companies
Margaret G. Ehm	GlaxoSmithKline, Collegeville, PA, United States	meg.g.ehm@gsk.com	Scientific Committee	Pharmaceutical companies
David Pulford	GlaxoSmithKline, Stevenage, United Kingdom	david.x.pulford@gsk.com	Scientific Committee	Pharmaceutical companies
Neha Raghavan	Merck, Kenilworth, NJ, United States	neha.raghavan@merck.com	Scientific Committee	Pharmaceutical companies
Adriana Huertas-Vazquez	Merck, Kenilworth, NJ, United States	adriana.huertas.vazquez@merck.com	Scientific Committee	Pharmaceutical companies
Jae-Hoon Sul	Merck, Kenilworth, NJ, United States	jae.hoon.sul@merck.com	Scientific Committee	Pharmaceutical companies
Anders Mälarstig	Pfizer, New York, NY, United States	anders.malarstig@pfizer.com	Scientific Committee	Pharmaceutical companies
Xinli Hu	Pfizer, New York, NY, United States	xinli.hu@pfizer.com	Scientific Committee	Pharmaceutical companies
Katherine Klinger	Translational Sciences, Sanofi R&D, Framingham, MA, USA	katherine.klinger@sanofi.com	Scientific Committee	Pharmaceutical companies
Robert Graham	Maze Therapeutics, San Francisco, CA, United States	rgraham@mazetx.com	Scientific Committee	Pharmaceutical companies
Eric Green	Maze Therapeutics, San Francisco, CA, United States	egreen@mazetx.com	Scientific Committee	Pharmaceutical companies
Sahar Mozaffari	Maze Therapeutics, San Francisco, CA, United States	smozaffari@mazetx.com	Scientific Committee	Pharmaceutical companies
Dawn Waterworth	Janssen Research & Development, LLC, Spring House, PA, United States	dwaterwo@its.jnj.com	Scientific Committee	Pharmaceutical companies
Nicole Renaud	Novartis Institutes for BioMedical Research, Cambridge, MA, United States	nicole.renaud@novartis.com	Scientific Committee	Pharmaceutical companies
Ma´en Obeidat	Novartis Institutes for BioMedical Research, Cambridge, MA, United States	maen.obeidat@novartis.com	Scientific Committee	Pharmaceutical companies
Samuli Ripatti	Institute for Molecular Medicine Finland (FIMM), HiLIFE, University of Helsinki, Helsinki, Finland	samuli.ripatti@helsinki.fi	Scientific Committee	University of Helsinki & Biobanks
Johanna Schleutker	Auria Biobank / Univ. of Turku / Hospital District of Southwest Finland, Turku, Finland	johanna.schleutker@utu.fi	Scientific Committee	University of Helsinki & Biobanks
Markus Perola	THL Biobank / Finnish Institute for Health and Welfare (THL), Helsinki, Finland	markus.perola@thl.fi	Scientific Committee	University of Helsinki & Biobanks
Mikko Arvas	Finnish Red Cross Blood Service / Finnish Hematology Registry and Clinical Biobank, Helsinki, Finland	mikko.arvas@veripalvelu.fi	Scientific Committee	University of Helsinki & Biobanks
Olli Carpén	Helsinki Biobank / Helsinki University and Hospital District of Helsinki and Uusimaa, Helsinki	olli.carpen@helsinki.fi	Scientific Committee	University of Helsinki & Biobanks
Reetta Hinttala	Northern Finland Biobank Borealis / University of Oulu / Northern Ostrobothnia Hospital District, Oulu, Finland	reetta.hinttala@oulu.fi	Scientific Committee	University of Helsinki & Biobanks
Johannes Kettunen	Northern Finland Biobank Borealis / University of Oulu / Northern Ostrobothnia Hospital District, Oulu, Finland	johannes.kettunen@oulu.fi	Scientific Committee	University of Helsinki & Biobanks
Arto Mannermaa	Biobank of Eastern Finland / University of Eastern Finland / Northern Savo Hospital District, Kuopio, Finland	arto.mannermaa@uef.fi	Scientific Committee	University of Helsinki & Biobanks
Katriina Aalto-Setälä	Faculty of Medicine and Health Technology, Tampere University, Tampere, Finland	katriina.aalto-setala@tuni.fi	Scientific Committee	University of Helsinki & Biobanks
Mika Kähönen	Finnish Clinical Biobank Tampere / University of Tampere / Pirkanmaa Hospital District, Tampere, Finland	mika.kahonen@uta.fi	Scientific Committee	University of Helsinki & Biobanks
Jari Laukkanen	Central Finland Biobank / University of Jyväskylä / Central Finland Health Care District, Jyväskylä, Finland	jari.laukkanen@ksshp.fi	Scientific Committee	University of Helsinki & Biobanks
Johanna Mäkelä	FINBB - Finnish biobank cooperative	johanna.makela@finbb.fi	Scientific Committee	University of Helsinki & Biobanks
Reetta Kälviäinen	Northern Savo Hospital District, Kuopio, Finland	reetta.kalviainen@kuh.fi	Clinical Groups	Neurology Group
Valtteri Julkunen	Northern Savo Hospital District, Kuopio, Finland	valtteri.julkunen@kuh.fi	Clinical Groups	Neurology Group
Hilkka Soininen	Northern Savo Hospital District, Kuopio, Finland	hilkka.soininen@uef.fi	Clinical Groups	Neurology Group
Anne Remes	Northern Ostrobothnia Hospital District, Oulu, Finland	anne.remes@oulu.fi	Clinical Groups	Neurology Group
Mikko Hiltunen	University of Eastern Finland, Kuopio, Finland	mikko.hiltunen@uef.fi	Clinical Groups	Neurology Group
Jukka Peltola	Pirkanmaa Hospital District, Tampere, Finland	jukka.peltola@pshp.fi	Clinical Groups	Neurology Group
Minna Raivio	Hospital District of Helsinki and Uusimaa, Helsinki, Finland	minna.raivio@geri.fi	Clinical Groups	Neurology Group
Pentti Tienari	Hospital District of Helsinki and Uusimaa, Helsinki, Finland	pentti.tienari@hus.fi	Clinical Groups	Neurology Group
Juha Rinne	Hospital District of Southwest Finland, Turku, Finland	juha.rinne@tyks.fi	Clinical Groups	Neurology Group
Roosa Kallionpää	Hospital District of Southwest Finland, Turku, Finland	roosa.kallionpaa@tyks.fi	Clinical Groups	Neurology Group
Juulia Partanen	Institute for Molecular Medicine Finland, HiLIFE, University of Helsinki, Finland	juulia.partanen@helsinki.fi	Clinical Groups	Neurology Group
Ali Abbasi	Abbvie, Chicago, IL, United States	ali.abbasi@abbvie.com	Clinical Groups	Neurology Group
Adam Ziemann	Abbvie, Chicago, IL, United States	adam.ziemann@abbvie.com	Clinical Groups	Neurology Group
Nizar Smaoui	Abbvie, Chicago, IL, United States	nizar.smaoui@abbvie.com	Clinical Groups	Neurology Group
Anne Lehtonen	Abbvie, Chicago, IL, United States	anne.lehtonen@abbvie.com	Clinical Groups	Neurology Group
Susan Eaton	Biogen, Cambridge, MA, United States	susan.eaton@biogen.com	Clinical Groups	Neurology Group
Heiko Runz	Biogen, Cambridge, MA, United States	heiko.runz@biogen.com	Clinical Groups	Neurology Group
Sanni Lahdenperä	Biogen, Cambridge, MA, United States	sanni.lahdenpera@biogen.com	Clinical Groups	Neurology Group
Shameek Biswas	Bristol Myers Squibb, New York, NY, United States	shameek.biswas@bms.com	Clinical Groups	Neurology Group
Julie Hunkapiller	Genentech, San Francisco, CA, United States	hunkapiller.julie@gene.com	Clinical Groups	Neurology Group
Natalie Bowers	Genentech, San Francisco, CA, United States	bowersn1@gene.com	Clinical Groups	Neurology Group
Edmond Teng	Genentech, San Francisco, CA, United States	teng.edmond@gene.com	Clinical Groups	Neurology Group
Rion Pendergrass	Genentech, San Francisco, CA, United States	penders2@gene.com	Clinical Groups	Neurology Group

Fanli Xu	GlaxoSmithKline, Brentford, United Kingdom	chun-fang.2.xu@gsk.com	Clinical Groups	Neurology Group
David Pulford	GlaxoSmithKline, Stevenage, United Kingdom	david.x.pulford@gsk.com	Clinical Groups	Neurology Group
Kirsi Auro	GlaxoSmithKline, Espoo, Finland	kirsi.m.auro@gsk.com	Clinical Groups	Neurology Group
Laura Addis	GlaxoSmithKline, Brentford, United Kingdom	laura.x.addis@gsk.com	Clinical Groups	Neurology Group
John Eicher	GlaxoSmithKline, Brentford, United Kingdom	john.d.eicher@gsk.com	Clinical Groups	Neurology Group
Qingqin S Li	Janssen Research & Development, LLC, Titusville, NJ 08560, United States	QLi2@its.jnj.com	Clinical Groups	Neurology Group
Karen He	Janssen Research & Development, LLC, Spring House, PA, United States	khe2@its.jnj.com	Clinical Groups	Neurology Group
Ekaterina Khramtsova	Janssen Research & Development, LLC, Spring House, PA, United States	ekhramts@its.jnj.com	Clinical Groups	Neurology Group
Neha Raghavan	Merck, Kenilworth, NJ, United States	neha.raghavan@merck.com	Clinical Groups	Neurology Group
Martti Färkkilä	Hospital District of Helsinki and Uusimaa, Helsinki, Finland	martti.farkkila@hus.fi	Clinical Groups	Gastroenterology Group
Jukka Koskela	Hospital District of Helsinki and Uusimaa, Helsinki, Finland	jukka.koskela@helsinki.fi	Clinical Groups	Gastroenterology Group
Sampsa Pikkarainen	Hospital District of Helsinki and Uusimaa, Helsinki, Finland	sampsa.pikkarainen@hus.fi	Clinical Groups	Gastroenterology Group
Airi Jussila	Pirkanmaa Hospital District, Tampere, Finland	airi.jussila@pshp.fi	Clinical Groups	Gastroenterology Group
Katri Kaukinen	Pirkanmaa Hospital District, Tampere, Finland	katri.kaukinen@tuni.fi	Clinical Groups	Gastroenterology Group
Timo Blomster	Northern Ostrobothnia Hospital District, Oulu, Finland	timo.blomster@ppshp.fi	Clinical Groups	Gastroenterology Group
Mikko Kiviniemi	Northern Savo Hospital District, Kuopio, Finland	mikko.kiviniemi@kuh.fi	Clinical Groups	Gastroenterology Group
Markku Voutilainen	Hospital District of Southwest Finland, Turku, Finland	markku.voutilainen@tyks.fi	Clinical Groups	Gastroenterology Group
Mark Daly	Institute for Molecular Medicine, Finland (FIMM), HiLIFE, University of Helsinki, Helsinki, Finland; Broad Institute of MIT and Harvard; Massachusetts General Hospital	mark.daly@helsinki.fi	Clinical Groups	Gastroenterology Group
Ali Abbasi	Abbvie, Chicago, IL, United States	ali.abbasi@abbvie.com	Clinical Groups	Gastroenterology Group
Jeffrey Waring	Abbvie, Chicago, IL, United States	jeff.waring@abbvie.com	Clinical Groups	Gastroenterology Group
Nizar Smaoui	Abbvie, Chicago, IL, United States	nizar.smaoui@abbvie.com	Clinical Groups	Gastroenterology Group
Fedik Rahimov	Abbvie, Chicago, IL, United States	fedik.rahimov@abbvie.com	Clinical Groups	Gastroenterology Group
Anne Lehtonen	Abbvie, Chicago, IL, United States	anne.lehtonen@abbvie.com	Clinical Groups	Gastroenterology Group
Tim Lu	Genentech, San Francisco, CA, United States	lut8@gene.com	Clinical Groups	Gastroenterology Group
Natalie Bowers	Genentech, San Francisco, CA, United States	bowersn1@gene.com	Clinical Groups	Gastroenterology Group
Rion Pendergrass	Genentech, San Francisco, CA, United States	penders2@gene.com	Clinical Groups	Gastroenterology Group
Linda McCarthy	GlaxoSmithKline, Brentford, United Kingdom	linda.c.mccarthy@gsk.com	Clinical Groups	Gastroenterology Group
Amy Hart	Janssen Research & Development, LLC, Spring House, PA, United States	ahart13@its.jnj.com	Clinical Groups	Gastroenterology Group
Meijian Guan	Janssen Research & Development, LLC, Spring House, PA, United States	mguan4@its.jnj.com	Clinical Groups	Gastroenterology Group
Jason Miller	Merck, Kenilworth, NJ, United States	jason.miller4@merck.com	Clinical Groups	Gastroenterology Group
Kirsi Kalpala	Pfizer, New York, NY, United States	kirsi.kalpala@pfizer.com	Clinical Groups	Gastroenterology Group
Melissa Miller	Pfizer, New York, NY, United States	melissa.r.miller@pfizer.com	Clinical Groups	Gastroenterology Group
Xinli Hu	Pfizer, New York, NY, United States	xinli.hu@pfizer.com	Clinical Groups	Gastroenterology Group
Kari Eklund	Hospital District of Helsinki and Uusimaa, Helsinki, Finland	kari.eklund@hus.fi	Clinical Groups	Rheumatology Group
Antti Palomäki	Hospital District of Southwest Finland, Turku, Finland	ajpalo@utu.fi	Clinical Groups	Rheumatology Group
Pia Isomäki	Pirkanmaa Hospital District, Tampere, Finland	pia.isomaki@pshp.fi	Clinical Groups	Rheumatology Group
Laura Pirilä	Hospital District of Southwest Finland, Turku, Finland	laura.pirila@fimnet.fi,laura.pirila@tyks.fi	Clinical Groups	Rheumatology Group
Oili Kaipiainen-Seppänen	Northern Savo Hospital District, Kuopio, Finland	oili.kaipiainen-seppanen@kuh.fi	Clinical Groups	Rheumatology Group
Johanna Huhtakangas	Northern Ostrobothnia Hospital District, Oulu, Finland	johanna.huhtakangas@kuh.fi	Clinical Groups	Rheumatology Group
Nina Mars	Institute for Molecular Medicine Finland (FIMM), HiLIFE, University of Helsinki, Helsinki, Finland	nina.mars@helsinki.fi	Clinical Groups	Rheumatology Group
Ali Abbasi	Abbvie, Chicago, IL, United States	ali.abbasi@abbvie.com	Clinical Groups	Rheumatology Group
Jeffrey Waring	Abbvie, Chicago, IL, United States	jeff.waring@abbvie.com	Clinical Groups	Rheumatology Group
Fedik Rahimov	Abbvie, Chicago, IL, United States	fedik.rahimov@abbvie.com	Clinical Groups	Rheumatology Group
Apinya Lertratanakul	Abbvie, Chicago, IL, United States	apinya.lertratanakul@abbvie.com	Clinical Groups	Rheumatology Group
Nizar Smaoui	Abbvie, Chicago, IL, United States	nizar.smaoui@abbvie.com	Clinical Groups	Rheumatology Group
Anne Lehtonen	Abbvie, Chicago, IL, United States	anne.lehtonen@abbvie.com	Clinical Groups	Rheumatology Group
Marla Hochfeld	Bristol Myers Squibb, New York, NY, United States	mhochfeld@celgene.com	Clinical Groups	Rheumatology Group
Natalie Bowers	Genentech, San Francisco, CA, United States	bowersn1@gene.com	Clinical Groups	Rheumatology Group
Rion Pendergrass	Genentech, San Francisco, CA, United States	penders2@gene.com	Clinical Groups	Rheumatology Group
Jorge Esparza Gordillo	GlaxoSmithKline, Brentford, United Kingdom	jorge.x.esparza-gordillo@gsk.com	Clinical Groups	Rheumatology Group
Kirsi Auro	GlaxoSmithKline, Espoo, Finland	kirsi.m.auro@gsk.com	Clinical Groups	Rheumatology Group
Dawn Waterworth	Janssen Research & Development, LLC, Spring House, PA, United States	dwaterwo@its.jnj.com	Clinical Groups	Rheumatology Group
Fabiana Farias	Merck, Kenilworth, NJ, United States	fabiana.farias@merck.com	Clinical Groups	Rheumatology Group
Kirsi Kalpala	Pfizer, New York, NY, United States	kirsi.kalpala@pfizer.com	Clinical Groups	Rheumatology Group
Nan Bing	Pfizer, New York, NY, United States	nan.bing@pfizer.com	Clinical Groups	Rheumatology Group
Xinli Hu	Pfizer, New York, NY, United States	xinli.hu@pfizer.com	Clinical Groups	Rheumatology Group
Tarja Laitinen	Pirkanmaa Hospital District, Tampere, Finland	tarja.laitinen@pshp.fi	Clinical Groups	Pulmonology Group

Margit Pelkonen	Northern Savo Hospital District, Kuopio, Finland	margit.pelkonen@kuh.fi	Clinical Groups	Pulmonology Group
Paula Kauppi	Hospital District of Helsinki and Uusimaa, Helsinki, Finland	paula.kauppi@hus.fi	Clinical Groups	Pulmonology Group
Hannu Kankaanranta	University of Gothenburg, Gothenburg, Sweden/ Seinäjoki Central Hospital, Seinäjoki, Finland/ Tampere University, Tampere, Finland	hannu.kankaanranta@tuni.fi	Clinical Groups	Pulmonology Group
Terttu Harju	Northern Ostrobothnia Hospital District, Oulu, Finland	terttu.harju@oulu.fi	Clinical Groups	Pulmonology Group
Riitta Lahesmaa	Hospital District of Southwest Finland, Turku, Finland	rilahes@utu.fi	Clinical Groups	Pulmonology Group
Nizar Smaoui	Abbvie, Chicago, IL, United States	nizar.smaoui@abbvie.com	Clinical Groups	Pulmonology Group
Glenda Lassi	Astra Zeneca, Cambridge, United Kingdom	glenda.lassi@astrazeneca.com	Clinical Groups	Pulmonology Group
Susan Eaton	Biogen, Cambridge, MA, United States	susan.eaton@biogen.com	Clinical Groups	Pulmonology Group
Hubert Chen	Genentech, San Francisco, CA, United States	chenh37@gene.com	Clinical Groups	Pulmonology Group
Rion Pendergrass	Genentech, San Francisco, CA, United States	penders2@gene.com	Clinical Groups	Pulmonology Group
Natalie Bowers	Genentech, San Francisco, CA, United States	bowersn1@gene.com	Clinical Groups	Pulmonology Group
Joanna Betts	GlaxoSmithKline, Brentford, United Kingdom	joanna.c.betts@gsk.com	Clinical Groups	Pulmonology Group
Kirsi Auro	GlaxoSmithKline, Espoo, Finland	kirsi.m.auro@gsk.com	Clinical Groups	Pulmonology Group
Rajashree Mishra	GlaxoSmithKline, Brentford, United Kingdom	rajashree.x.mishra@gsk.com	Clinical Groups	Pulmonology Group
Majd Mouded	Novartis, Basel, Switzerland	majd.mouded@novartis.com	Clinical Groups	Pulmonology Group
Debby Ngo	Novartis, Basel, Switzerland	debby.ngo@novartis.com	Clinical Groups	Pulmonology Group
Teemu Niiranen	Finnish Institute for Health and Welfare (THL), Helsinki, Finland	teemu.niiranen@thl.fi	Clinical Groups	Cardiometabolic Diseases Group
Felix Vaura	Finnish Institute for Health and Welfare (THL), Helsinki, Finland	fechva@utu.fi	Clinical Groups	Cardiometabolic Diseases Group
Veikko Salomaa	Finnish Institute for Health and Welfare (THL), Helsinki, Finland	veikko.salomaa@thl.fi	Clinical Groups	Cardiometabolic Diseases Group
Kaj Metsärinne	Hospital District of Southwest Finland, Turku, Finland	kaj.metsarinne@tyks.fi	Clinical Groups	Cardiometabolic Diseases Group
Jenni Aittokallio	Hospital District of Southwest Finland, Turku, Finland	jemato@utu.fi	Clinical Groups	Cardiometabolic Diseases Group
Mika Kähönen	Pirkanmaa Hospital District, Tampere, Finland	mika.kahonen@uta.fi	Clinical Groups	Cardiometabolic Diseases Group
Jussi Hernesniemi	Pirkanmaa Hospital District, Tampere, Finland	jussi.hernesniemi@tuni.fi	Clinical Groups	Cardiometabolic Diseases Group
Daniel Gordin	Hospital District of Helsinki and Uusimaa, Helsinki, Finland	daniel.gordin@hus.fi	Clinical Groups	Cardiometabolic Diseases Group
Juha Sinisalo	Hospital District of Helsinki and Uusimaa, Helsinki, Finland	juha.sinisalo@hus.fi	Clinical Groups	Cardiometabolic Diseases Group
Marja-Riitta Taskinen	Hospital District of Helsinki and Uusimaa, Helsinki, Finland	marja-riitta.taskinen@helsinki.fi	Clinical Groups	Cardiometabolic Diseases Group
Tiinamaija Tuomi	Hospital District of Helsinki and Uusimaa, Helsinki, Finland	tiinamaija.tuomi@hus.fi	Clinical Groups	Cardiometabolic Diseases Group
Timo Hiltunen	Hospital District of Helsinki and Uusimaa, Helsinki, Finland	timo.hiltunen@hus.fi	Clinical Groups	Cardiometabolic Diseases Group
Jari Laukkanen	Central Finland Health Care District, Jyväskylä, Finland	jari.laukkanen@ksshp.fi	Clinical Groups	Cardiometabolic Diseases Group
Amanda Elliott	Institute for Molecular Medicine Finland (FIMM), HiLIFE, University of Helsinki, Helsinki, Finland; Broad Institute, Cambridge, MA, USA and Massachusetts General Hospital, Boston, MA, USA	aelliott@broadinstitute.org	Clinical Groups	Cardiometabolic Diseases Group
Mary Pat Reeve	Institute for Molecular Medicine Finland (FIMM), HiLIFE, University of Helsinki, Helsinki, Finland	mary.reeve@helsinki.fi	Clinical Groups	Cardiometabolic Diseases Group
Sanni Ruotsalainen	Institute for Molecular Medicine Finland (FIMM), HiLIFE, University of Helsinki, Helsinki, Finland	sanni.ruotsalainen@helsinki.fi	Clinical Groups	Cardiometabolic Diseases Group
Benjamin Challis	Astra Zeneca, Cambridge, United Kingdom	benjamin.challis@astrazeneca.com	Clinical Groups	Cardiometabolic Diseases Group
Dirk Paul	Astra Zeneca, Cambridge, United Kingdom	dirk.paul@astrazeneca.com	Clinical Groups	Cardiometabolic Diseases Group
Julie Hunkapiller	Genentech, San Francisco, CA, United States	hunkapiller.julie@gene.com	Clinical Groups	Cardiometabolic Diseases Group
Natalie Bowers	Genentech, San Francisco, CA, United States	bowersn1@gene.com	Clinical Groups	Cardiometabolic Diseases Group
Rion Pendergrass	Genentech, San Francisco, CA, United States	penders2@gene.com	Clinical Groups	Cardiometabolic Diseases Group
Audrey Chu	GlaxoSmithKline, Brentford, United Kingdom	audrey.y.chu@gsk.com	Clinical Groups	Cardiometabolic Diseases Group
Kirsi Auro	GlaxoSmithKline, Espoo, Finland	kirsi.m.auro@gsk.com	Clinical Groups	Cardiometabolic Diseases Group
Dermot Reilly	Janssen Research & Development, LLC, Boston, MA, United States	dreill11@its.jnj.com	Clinical Groups	Cardiometabolic Diseases Group
Mike Mendelson	Novartis, Boston, MA, United States	mike.mendelson@novartis.com	Clinical Groups	Cardiometabolic Diseases Group
Jaakko Parkkinen	Pfizer, New York, NY, United States	jaakko.parkkinen@pfizer.com	Clinical Groups	Cardiometabolic Diseases Group
Melissa Miller	Pfizer, New York, NY, United States	melissa.r.miller@pfizer.com	Clinical Groups	Cardiometabolic Diseases Group
Tuomo Meretoja	Hospital District of Helsinki and Uusimaa, Helsinki, Finland	tuomo.meretoja@hus.fi	Clinical Groups	Oncology Group
Heikki Joensuu	Hospital District of Helsinki and Uusimaa, Helsinki, Finland	heikki.joensuu@hus.fi	Clinical Groups	Oncology Group
Olli Carpén	Hospital District of Helsinki and Uusimaa, Helsinki, Finland	olli.carpen@helsinki.fi	Clinical Groups	Oncology Group
Johanna Mattson	Hospital District of Helsinki and Uusimaa, Helsinki, Finland	johanna.mattson@hus.fi	Clinical Groups	Oncology Group
Eveliina Salminen	Hospital District of Helsinki and Uusimaa, Helsinki, Finland	eveliina.e.salminen@hus.fi	Clinical Groups	Oncology Group
Annika Auranen	Pirkanmaa Hospital District , Tampere, Finland	anaura@utu.fi	Clinical Groups	Oncology Group
Peeter Karihtala	Northern Ostrobothnia Hospital District, Oulu, Finland	peeter.karihtala@oulu.fi	Clinical Groups	Oncology Group
Päivi Auvinen	Northern Savo Hospital District, Kuopio, Finland	paivi.auvinen@kuh.fi	Clinical Groups	Oncology Group
Klaus Elenius	Hospital District of Southwest Finland, Turku, Finland	klaus.elenius@utu.fi	Clinical Groups	Oncology Group
Johanna Schleutker	Hospital District of Southwest Finland, Turku, Finland	johanna.schleutker@utu.fi	Clinical Groups	Oncology Group
Esa Pitkänen	Institute for Molecular Medicine Finland (FIMM), HiLIFE, University of Helsinki, Helsinki, Finland	esa.pitkanen@helsinki.fi	Clinical Groups	Oncology Group
Nina Mars	Institute for Molecular Medicine Finland (FIMM), HiLIFE, University of Helsinki, Helsinki, Finland	nina.mars@helsinki.fi	Clinical Groups	Oncology Group
Mark Daly	Institute for Molecular Medicine Finland (FIMM), HiLIFE, University of Helsinki, Helsinki, Finland; Broad Institute of MIT and Harvard; Massachusetts General Hospital	mark.daly@helsinki.fi	Clinical Groups	Oncology Group
Relja Popovic	Abbvie, Chicago, IL, United States	relja.popovic@abbvie.com	Clinical Groups	Oncology Group
Jeffrey Waring	Abbvie, Chicago, IL, United States	jeff.waring@abbvie.com	Clinical Groups	Oncology Group
Bridget Riley-Gillis	Abbvie, Chicago, IL, United States	bridget.rileygillis@abbvie.com	Clinical Groups	Oncology Group

Anne Lehtonen	Abbvie, Chicago, IL, United States	anne.lehtonen@abbvie.com	Clinical Groups	Oncology Group
Jennifer Schutzman	Genentech, San Francisco, CA, United States	schutzman.jennifer@gene.com	Clinical Groups	Oncology Group
Julie Hunkapiller	Genentech, San Francisco, CA, United States	hunkapiller.julie@gene.com	Clinical Groups	Oncology Group
Natalie Bowers	Genentech, San Francisco, CA, United States	bowersn1@gene.com	Clinical Groups	Oncology Group
Rion Pendergrass	Genentech, San Francisco, CA, United States	penders2@gene.com	Clinical Groups	Oncology Group
Diptee Kulkarni	GlaxoSmithKline, Brentford, United Kingdom	diptee.a.kulkarni@gsk.com	Clinical Groups	Oncology Group
Kirsi Auro	GlaxoSmithKline, Espoo, Finland	kirsi.m.auro@gsk.com	Clinical Groups	Oncology Group
Alessandro Porello	Janssen Research & Development, LLC, Spring House, PA, United States	APorrell@ITS.JNJ.com	Clinical Groups	Oncology Group
Andrey Loboda	Merck, Kenilworth, NJ, United States	andrey_loboda@merck.com	Clinical Groups	Oncology Group
Heli Lehtonen	Pfizer, New York, NY, United States	heli.lehtonen@pfizer.com	Clinical Groups	Oncology Group
Stefan McDonough	Pfizer, New York, NY, United States	stefan.McDonough@pfizer.com	Clinical Groups	Oncology Group
Sauli Vuoti	Janssen-Cilag Oy, Espoo, Finland	svuoti@its.jnj.com	Clinical Groups	Oncology Group
Kai Kaarniranta	Northern Savo Hospital District, Kuopio, Finland	kai.kaarniranta@uef.fi	Clinical Groups	Opthalmology Group
Joni A Turunen	Helsinki University Hospital and University of Helsinki, Helsinki, Finland; Eye Genetics Group, Folkhälsan Research Center, Helsinki, Finland	joni.turunen@helsinki.fi	Clinical Groups	Opthalmology Group
Terhi Ollila	Hospital District of Helsinki and Uusimaa, Helsinki, Finland	terhi.ollila@hus.fi	Clinical Groups	Opthalmology Group
Hannu Uusitalo	Pirkanmaa Hospital District, Tampere, Finland	hannu.uusitalo@tuni.fi	Clinical Groups	Opthalmology Group
Juha Karjalainen	Institute for Molecular Medicine Finland (FIMM), HiLIFE, University of Helsinki, Helsinki, Finland	juha.karjalainen@helsinki.fi	Clinical Groups	Opthalmology Group
Esa Pitkänen	Institute for Molecular Medicine Finland (FIMM), HiLIFE, University of Helsinki, Helsinki, Finland	esa.pitkanen@helsinki.fi	Clinical Groups	Opthalmology Group
Mengzhen Liu	Abbvie, Chicago, IL, United States	mengzhen.liu@abbvie.com	Clinical Groups	Opthalmology Group
Heiko Runz	Biogen, Cambridge, MA, United States	heiko.runz@biogen.com	Clinical Groups	Opthalmology Group
Stephanie Loomis	Biogen, Cambridge, MA, United States	stephanie.loomis@biogen.com	Clinical Groups	Opthalmology Group
Erich Strauss	Genentech, San Francisco, CA, United States	strauss.erich@gene.com	Clinical Groups	Opthalmology Group
Natalie Bowers	Genentech, San Francisco, CA, United States	bowersn1@gene.com	Clinical Groups	Opthalmology Group
Hao Chen	Genentech, San Francisco, CA, United States	haoc@gene.com	Clinical Groups	Opthalmology Group
Rion Pendergrass	Genentech, San Francisco, CA, United States	penders2@gene.com	Clinical Groups	Opthalmology Group
Kaisa Tasanen	Northern Ostrobothnia Hospital District, Oulu, Finland	kaisa.tasanen-maatta@oulu.fi	Clinical Groups	Dermatology Group
Laura Huilaja	Northern Ostrobothnia Hospital District, Oulu, Finland	laura.huilaja@oulu.fi	Clinical Groups	Dermatology Group
Katariina Hannula-Jouppi	Hospital District of Helsinki and Uusimaa, Helsinki, Finland	katariina.hannula-jouppi@hus.fi	Clinical Groups	Dermatology Group
Teea Salmi	Pirkanmaa Hospital District, Tampere, Finland	teea.salmi@pshp.fi	Clinical Groups	Dermatology Group
Sirkku Peltonen	Hospital District of Southwest Finland, Turku, Finland	sipelto@utu.fi	Clinical Groups	Dermatology Group
Leena Koulu	Hospital District of Southwest Finland, Turku, Finland	leena.koulu@tyks.fi	Clinical Groups	Dermatology Group
Nizar Smaoui	Abbvie, Chicago, IL, United States	nizar.smaoui@abbvie.com	Clinical Groups	Dermatology Group
Fedik Rahimov	Abbvie, Chicago, IL, United States	fedik.rahimov@abbvie.com	Clinical Groups	Dermatology Group
Anne Lehtonen	Abbvie, Chicago, IL, United States	anne.lehtonen@abbvie.com	Clinical Groups	Dermatology Group
David Choy	Genentech, San Francisco, CA, United States	choy.david@gene.com	Clinical Groups	Dermatology Group
Rion Pendergrass	Genentech, San Francisco, CA, United States	penders2@gene.com	Clinical Groups	Dermatology Group
Dawn Waterworth	Janssen Research & Development, LLC, Spring House, PA, United States	dwaterwo@its.jnj.com	Clinical Groups	Dermatology Group
Kirsi Kalpala	Pfizer, New York, NY, United States	kirsi.kalpala@pfizer.com	Clinical Groups	Dermatology Group
Ying Wu	Pfizer, New York, NY, United States	ying.wu3@pfizer.com	Clinical Groups	Dermatology Group
Pirkko Pussinen	Hospital District of Helsinki and Uusimaa, Helsinki, Finland	pirkko.pussinen@helsinki.fi	Clinical Groups	Odontology Group
Aino Salminen	Hospital District of Helsinki and Uusimaa, Helsinki, Finland	aino.m.salminen@helsinki.fi	Clinical Groups	Odontology Group
Tuula Salo	Hospital District of Helsinki and Uusimaa, Helsinki, Finland	tuula.salo@helsinki.fi	Clinical Groups	Odontology Group
David Rice	Hospital District of Helsinki and Uusimaa, Helsinki, Finland	david.rice@helsinki.fi	Clinical Groups	Odontology Group
Pekka Nieminen	Hospital District of Helsinki and Uusimaa, Helsinki, Finland	pekka.nieminen@helsinki.fi	Clinical Groups	Odontology Group
Ulla Palotie	Hospital District of Helsinki and Uusimaa, Helsinki, Finland	ulla.palotie@helsinki.fi	Clinical Groups	Odontology Group
Maria Siponen	Northern Savo Hospital District, Kuopio, Finland	maria.siponen@uef.fi	Clinical Groups	Odontology Group
Liisa Suominen	Northern Savo Hospital District, Kuopio, Finland	liisa.suominen@uef.fi	Clinical Groups	Odontology Group
Päivi Mäntylä	Northern Savo Hospital District, Kuopio, Finland	paivi.mantyla@uef.fi	Clinical Groups	Odontology Group
Ulvi Gursoy	Hospital District of Southwest Finland, Turku, Finland	ulvi.gursoy@utu.fi	Clinical Groups	Odontology Group
Vuokko Anttonen	Northern Ostrobothnia Hospital District, Oulu, Finland	vuokko.anttonen@oulu.fi	Clinical Groups	Odontology Group
Kirsi Sipilä	Research Unit of Oral Health Sciences Faculty of Medicine, University of Oulu, Oulu, Finland; Medical Research Center, Oulu, Oulu University Hospital and University of Oulu, Oulu, Finland	kirsi.sipila@oulu.fi	Clinical Groups	Odontology Group
Rion Pendergrass	Genentech, San Francisco, CA, United States	pendergrass.sarah@gene.com	Clinical Groups	Odontology Group
Hannele Laivuori	Institute for Molecular Medicine Finland (FIMM), HiLIFE, University of Helsinki, Helsinki, Finland	hannele.laivuori@helsinki.fi	Clinical Groups	Women's Health and Reproduction Group
Venla Kurra	Pirkanmaa Hospital District, Tampere, Finland	venla.kurra@tuni.fi	Clinical Groups	Women's Health and Reproduction Group
Laura Kotaniemi-Talonen	Pirkanmaa Hospital District, Tampere, Finland	laura.kotaniemi-talonen@tuni.fi	Clinical Groups	Women's Health and Reproduction Group
Oskari Heikinheimo	Hospital District of Helsinki and Uusimaa, Helsinki, Finland	oskari.heikinheimo@helsinki.fi	Clinical Groups	Women's Health and Reproduction Group
Ilkka Kalliala	Hospital District of Helsinki and Uusimaa, Helsinki, Finland	ilkka.kalliala@hus.fi	Clinical Groups	Women's Health and Reproduction Group
Lauri Aaltonen	Hospital District of Helsinki and Uusimaa, Helsinki, Finland	lauri.aaltonen@helsinki.fi	Clinical Groups	Women's Health and Reproduction Group
Varpu Jokimaa	Hospital District of Southwest Finland, Turku, Finland	varpu.jokimaa@utu.fi	Clinical Groups	Women's Health and Reproduction Group
Johannes Kettunen	Northern Ostrobothnia Hospital District, Oulu, Finland	Johannes.Kettunen@oulu.fi	Clinical Groups	Women's Health and Reproduction Group

Marja Vääräsmäki	Northern Ostrobothnia Hospital District, Oulu, Finland	marja.vaarasmaki@oulu.fi	Clinical Groups	Women's Health and Reproduction Group
Outi Uimari	Northern Ostrobothnia Hospital District, Oulu, Finland	outi.uimari@oulu.fi	Clinical Groups	Women's Health and Reproduction Group
Laure Morin-Papunen	Northern Ostrobothnia Hospital District, Oulu, Finland	lmp@cc.oulu.fi	Clinical Groups	Women's Health and Reproduction Group
Maarit Niinimäki	Northern Ostrobothnia Hospital District, Oulu, Finland	maarit.niinimaki@oulu.fi	Clinical Groups	Women's Health and Reproduction Group
Terhi Piltonen	Northern Ostrobothnia Hospital District, Oulu, Finland	terhi.piltonen@oulu.fi	Clinical Groups	Women's Health and Reproduction Group
Katja Kivinen	Institute for Molecular Medicine Finland (FIMM), HiLIFE, University of Helsinki, Helsinki, Finland	katja.kivinen@helsinki.fi	Clinical Groups	Women's Health and Reproduction Group
Elisabeth Widen	Institute for Molecular Medicine Finland (FIMM), HiLIFE, University of Helsinki, Helsinki, Finland	elisabeth.widen@helsinki.fi	Clinical Groups	Women's Health and Reproduction Group
Taru Tukiainen	Institute for Molecular Medicine Finland (FIMM), HiLIFE, University of Helsinki, Helsinki, Finland	taru.tukiainen@helsinki.fi	Clinical Groups	Women's Health and Reproduction Group
Mary Pat Reeve	Institute for Molecular Medicine Finland (FIMM), HiLIFE, University of Helsinki, Helsinki, Finland	mary.reeve@helsinki.fi	Clinical Groups	Women's Health and Reproduction Group
Mark Daly	Institute for Molecular Medicine Finland (FIMM), HiLIFE, University of Helsinki, Helsinki, Finland; Broad Institute of MIT and Harvard; Massachusetts General Hospital	mark.daly@helsinki.fi	Clinical Groups	Women's Health and Reproduction Group
Niko Välimäki	University of Helsinki, Helsinki, Finland	niko.valimaki@helsinki.fi	Clinical Groups	Women's Health and Reproduction Group
Eija Laakkonen	University of Jyväskylä, Jyväskylä, Finland	eija.k.laakkonen@jyu.fi	Clinical Groups	Women's Health and Reproduction Group
Jaakko Tyrmi	University of Oulu, Oulu, Finland / University of Tampere, Tampere, Finland	jaakko.tyrmi@oulu.fi	Clinical Groups	Women's Health and Reproduction Group
Heidi Silven	University of Oulu, Oulu, Finland	heidi.silven@student.oulu.fi	Clinical Groups	Women's Health and Reproduction Group
Eeva Sliz	University of Oulu, Oulu, Finland	eeva.sliz@oulu.fi	Clinical Groups	Women's Health and Reproduction Group
Riikka Arffman	University of Oulu, Oulu, Finland	riikka.arffman@oulu.fi	Clinical Groups	Women's Health and Reproduction Group
Susanna Savukoski	University of Oulu, Oulu, Finland	susanna.savukoski@oulu.fi	Clinical Groups	Women's Health and Reproduction Group
Triin Laisk	Estonian biobank, Tartu, Estonia	triin.laisk@ut.ee	Clinical Groups	Women's Health and Reproduction Group
Natalia Pujol	Estonian biobank, Tartu, Estonia	natalia.pujolgualdo@oulu.fi	Clinical Groups	Women's Health and Reproduction Group
Mengzhen Liu	Abbvie, Chicago, IL, United States	mengzhen.liu@abbvie.com	Clinical Groups	Women's Health and Reproduction Group
Bridget Riley-Gillis	Abbvie, Chicago, IL, United States	bridget.rileygillis@abbvie.com	Clinical Groups	Women's Health and Reproduction Group
Rion Pendergrass	Genentech, San Francisco, CA, United States	penders2@gene.com	Clinical Groups	Women's Health and Reproduction Group
Janet Kumar	GlaxoSmithKline, Collegeville, PA, United States	janet.x.kumar@gsk.com	Clinical Groups	Women's Health and Reproduction Group
Kirsi Auro	GlaxoSmithKline, Espoo, Finland	kirsi.m.auro@gsk.com	Clinical Groups	Women's Health and Reproduction Group
Iiris Hovatta	University of Helsinki, Finland	iiris.hovatta@helsinki.fi	Clinical Groups	Depression group
Chia-Yen Chen	Biogen, Cambridge, MA, United States	chiayen.chen@biogen.com	Clinical Groups	Depression group
Erkki Isometsä	Hospital District of Helsinki and Uusimaa, Helsinki, Finland	erkki.isometsa@hus.fi	Clinical Groups	Depression group
Hanna Ollila	Institute for Molecular Medicine Finland (FIMM), HiLIFE, University of Helsinki, Helsinki, Finland	hanna.m.ollila@helsinki.fi	Clinical Groups	Depression group
Jaana Suvisaari	Finnish Institute for Health and Welfare (THL), Helsinki, Finland	jaana.suvisaari@thl.fi	Clinical Groups	Depression group
Thomas Damm Als	Aarhus University, Denmark	tda@biomed.au.dk	Clinical Groups	Depression group
Antti Mäkitie	Department of Otorhinolaryngology - Head and Neck Surgery, University of Helsinki and Helsinki University Hospital, Helsinki, Finland	antti.makitie@helsinki.fi	Clinical Groups	ENT (ear, nose and throath) Group
Argyro Bizaki-Vallaskangas	Pirkanmaa Hospital District, Tampere, Finland	argyro.bizaki-vallaskangas@tuni.fi	Clinical Groups	ENT (ear, nose and throath) Group
Sanna Toppila-Salmi	University of Helsinki, Finland	sanna.salmi@helsinki.fi	Clinical Groups	ENT (ear, nose and throath) Group
Tytti Willberg	Hospital District of Southwest Finland, Turku, Finland	tytti.willberg@tyks.fi	Clinical Groups	ENT (ear, nose and throath) Group
Elmo Saarentaus	Institute for Molecular Medicine Finland (FIMM), HiLIFE, University of Helsinki, Helsinki, Finland	elmo.saarentaus@helsinki.fi	Clinical Groups	ENT (ear, nose and throath) Group
Antti Aarnisalo	Hospital District of Helsinki and Uusimaa, Helsinki, Finland	antti.aarnisalo@hus.fi	Clinical Groups	ENT (ear, nose and throath) Group
Eveliina Salminen	Hospital District of Helsinki and Uusimaa, Helsinki, Finland	eveliina.e.salminen@hus.fi	Clinical Groups	ENT (ear, nose and throath) Group
Elisa Rahikkala	Northern Ostrobothnia Hospital District, Oulu, Finland	elisa.rahikkala@ppshp.fi	Clinical Groups	ENT (ear, nose and throath) Group
Johannes Kettunen	Northern Ostrobothnia Hospital District, Oulu, Finland	johannes.kettunen@oulu.fi	Clinical Groups	ENT (ear, nose and throath) Group
Kristiina Aittomäki	Department of Medical Genetics, Helsinki University Central Hospital, Helsinki, Finland	kristiina.aittomaki@helsinki.fi	Clinical Groups	POI (premature ovarian failure) Group
Fredrik Åberg	Transplantation and Liver Surgery Clinic, Helsinki University Hospital, Helsinki University, Helsinki, Finland	fredrik.aberg@helsinki.fi	Clinical Groups	LiverScore Group
Mitja Kurki	Institute for Molecular Medicine Finland (FIMM), HiLIFE, University of Helsinki, Helsinki, Finland; Broad Institute, Cambridge, MA, United States	mkurki@broadinstitute.org	FinnGen Analysis working group	FinnGen Analysis working group
Samuli Ripatti	Institute for Molecular Medicine Finland (FIMM), HiLIFE, University of Helsinki, Helsinki, Finland	samuli.ripatti@helsinki.fi	FinnGen Analysis working group	FinnGen Analysis working group
Mark Daly	Institute for Molecular Medicine, Finland (FIMM), HiLIFE, University of Helsinki, Helsinki, Finland; Broad Institute of MIT and Harvard; Massachusetts General Hospital	mark.daly@helsinki.fi	FinnGen Analysis working group	FinnGen Analysis working group

Juha Karjalainen	Institute for Molecular Medicine Finland (FIMM), HiLIFE, University of Helsinki, Helsinki, Finland	juha.karjalainen@helsinki.fi	FinnGen Analysis working group	FinnGen Analysis working group
Aki Havulinna	Institute for Molecular Medicine Finland (FIMM), HiLIFE, University of Helsinki, Helsinki, Finland; Finnish Institute for Health and Welfare (THL), Helsinki, Finland	aki.havulinna@helsinki.fi	FinnGen Analysis working group	FinnGen Analysis working group
Juha Mehtonen	Institute for Molecular Medicine Finland (FIMM), HiLIFE, University of Helsinki, Helsinki, Finland	juha.mehtonen@helsinki.fi	FinnGen Analysis working group	FinnGen Analysis working group
Priit Palta	Institute for Molecular Medicine Finland (FIMM), HiLIFE, University of Helsinki, Helsinki, Finland	priit.palta@helsinki.fi	FinnGen Analysis working group	FinnGen Analysis working group
Shabbeer Hassan	Institute for Molecular Medicine Finland (FIMM), HiLIFE, University of Helsinki, Helsinki, Finland	shabbeer.hassan@helsinki.fi	FinnGen Analysis working group	FinnGen Analysis working group
Pietro Della Briotta Parolo	Institute for Molecular Medicine Finland (FIMM), HiLIFE, University of Helsinki, Helsinki, Finland	pietro.dellabriottaparolo@helsinki.fi	FinnGen Analysis working group	FinnGen Analysis working group
Wei Zhou	Broad Institute, Cambridge, MA, United States	wzhou@broadinstitute.org	FinnGen Analysis working group	FinnGen Analysis working group
Mutaamba Maasha	Broad Institute, Cambridge, MA, United States	mmaasha@broadinstitute.org	FinnGen Analysis working group	FinnGen Analysis working group
Shabbeer Hassan	Institute for Molecular Medicine Finland (FIMM), HiLIFE, University of Helsinki, Helsinki, Finland	shabbeer.hassan@helsinki.fi	FinnGen Analysis working group	FinnGen Analysis working group
Susanna Lemmelä	Institute for Molecular Medicine Finland (FIMM), HiLIFE, University of Helsinki, Helsinki, Finland	susanna.lemmela@helsinki.fi	FinnGen Analysis working group	FinnGen Analysis working group
Manuel Rivas	University of Stanford, Stanford, CA, United States	mrivas@stanford.edu	FinnGen Analysis working group	FinnGen Analysis working group
Mari E. Niemi	Institute for Molecular Medicine Finland (FIMM), HiLIFE, University of Helsinki, Helsinki, Finland	mari.e.niemi@helsinki.fi	FinnGen Analysis working group	FinnGen Analysis working group
Aarno Palotie	Institute for Molecular Medicine Finland (FIMM), HiLIFE, University of Helsinki, Helsinki, Finland	aarno.palotie@helsinki.fi	FinnGen Analysis working group	FinnGen Analysis working group
Aoxing Liu	Institute for Molecular Medicine Finland (FIMM), HiLIFE, University of Helsinki, Helsinki, Finland	aoxing.liu@helsinki.fi	FinnGen Analysis working group	FinnGen Analysis working group
Arto Lehisto	Institute for Molecular Medicine Finland (FIMM), HiLIFE, University of Helsinki, Helsinki, Finland	arto.lehisto@helsinki.fi	FinnGen Analysis working group	FinnGen Analysis working group
Andrea Ganna	Institute for Molecular Medicine Finland (FIMM), HiLIFE, University of Helsinki, Helsinki, Finland	aganna@broadinstitute.org	FinnGen Analysis working group	FinnGen Analysis working group
Vincent Llorens	Institute for Molecular Medicine Finland (FIMM), HiLIFE, University of Helsinki, Helsinki, Finland	vincent.llorens@helsinki.fi	FinnGen Analysis working group	FinnGen Analysis working group
Hannele Laivuori	Institute for Molecular Medicine Finland (FIMM), HiLIFE, University of Helsinki, Helsinki, Finland	hannele.laivuori@helsinki.fi	FinnGen Analysis working group	FinnGen Analysis working group
Taru Tukiainen	Institute for Molecular Medicine Finland (FIMM), HiLIFE, University of Helsinki, Helsinki, Finland	taru.tukiainen@helsinki.fi	FinnGen Analysis working group	FinnGen Analysis working group
Mary Pat Reeve	Institute for Molecular Medicine Finland (FIMM), HiLIFE, University of Helsinki, Helsinki, Finland	mary.reeve@helsinki.fi	FinnGen Analysis working group	FinnGen Analysis working group
Henrike Heyne	Institute for Molecular Medicine Finland (FIMM), HiLIFE, University of Helsinki, Helsinki, Finland	hheyne@broadinstitute.org	FinnGen Analysis working group	FinnGen Analysis working group
Nina Mars	Institute for Molecular Medicine Finland (FIMM), HiLIFE, University of Helsinki, Helsinki, Finland	nina.mars@helsinki.fi	FinnGen Analysis working group	FinnGen Analysis working group
Joel Rämö	Institute for Molecular Medicine Finland (FIMM), HiLIFE, University of Helsinki, Helsinki, Finland	joel.ramo@helsinki.fi	FinnGen Analysis working group	FinnGen Analysis working group
Elmo Saarentaus	Institute for Molecular Medicine Finland (FIMM), HiLIFE, University of Helsinki, Helsinki, Finland	elmo.saarentaus@helsinki.fi	FinnGen Analysis working group	FinnGen Analysis working group
Hanna Ollila	Institute for Molecular Medicine Finland (FIMM), HiLIFE, University of Helsinki, Helsinki, Finland	hanna.m.ollila@helsinki.fi	FinnGen Analysis working group	FinnGen Analysis working group
Rodos Rodosthenous	Institute for Molecular Medicine Finland (FIMM), HiLIFE, University of Helsinki, Helsinki, Finland	rodos.rodosthenous@helsinki.fi	FinnGen Analysis working group	FinnGen Analysis working group
Satu Strausz	Institute for Molecular Medicine Finland (FIMM), HiLIFE, University of Helsinki, Helsinki, Finland	satu.strausz@helsinki.fi	FinnGen Analysis working group	FinnGen Analysis working group
Tuula Palotie	University of Helsinki and Hospital District of Helsinki and Uusimaa, Helsinki, Finland	tuula.palotie@helsinki.fi	FinnGen Analysis working group	FinnGen Analysis working group
Kimmo Palin	University of Helsinki, Helsinki, Finland	kimmo.palin@helsinki.fi	FinnGen Analysis working group	FinnGen Analysis working group
Javier Garcia-Tabuenca	University of Tampere, Tampere, Finland	javier.graciatabuenca@tuni.fi	FinnGen Analysis working group	FinnGen Analysis working group
Harri Siirtola	University of Tampere, Tampere, Finland	harri.siirtola@tuni.fi	FinnGen Analysis working group	FinnGen Analysis working group
Tuomo Kiiskinen	Institute for Molecular Medicine Finland (FIMM), HiLIFE, University of Helsinki, Helsinki, Finland	tuomo.kiiskinen@helsinki.fi	FinnGen Analysis working group	FinnGen Analysis working group
Jiwoo Lee	Institute for Molecular Medicine Finland (FIMM), HiLIFE, University of Helsinki, Helsinki, Finland; Broad Institute, Cambridge, MA, United States	jiwoo.lee@helsinki.fi	FinnGen Analysis working group	FinnGen Analysis working group
Kristin Tsuo	Institute for Molecular Medicine Finland (FIMM), HiLIFE, University of Helsinki, Helsinki, Finland; Broad Institute, Cambridge, MA, United States	kristintsuo@fas.harvard.edu	FinnGen Analysis working group	FinnGen Analysis working group
Amanda Elliott	Institute for Molecular Medicine Finland (FIMM), HiLIFE, University of Helsinki, Helsinki, Finland; Broad Institute, Cambridge, MA, USA and Massachusetts General Hospital, Boston, MA, USA	aelliott@broadinstitute.org	FinnGen Analysis working group	FinnGen Analysis working group
Kati Kristiansson	THL Biobank / Finnish Institute for Health and Welfare (THL), Helsinki, Finland	kati.kristiansson@thl.fi	FinnGen Analysis working group	FinnGen Analysis working group
Mikko Arvas	Finnish Red Cross Blood Service / Finnish Hematology Registry and Clinical Biobank, Helsinki, Finland	mikko.arvas@veripalvelu.fi	FinnGen Analysis working group	FinnGen Analysis working group
Kati Hyvärinen	Finnish Red Cross Blood Service, Helsinki, Finland	kati.hyvarinen@veripalvelu.fi	FinnGen Analysis working group	FinnGen Analysis working group
Jarmo Ritari	Finnish Red Cross Blood Service, Helsinki, Finland	jarmo.ritari@veripalvelu.fi	FinnGen Analysis working group	FinnGen Analysis working group
Olli Carpén	Helsinki Biobank / Helsinki University and Hospital District of Helsinki and Uusimaa, Helsinki	olli.carpen@helsinki.fi	FinnGen Analysis working group	FinnGen Analysis working group
Johannes Kettunen	Northern Finland Biobank Borealis / University of Oulu / Northern Ostrobothnia Hospital District, Oulu, Finland	johannes.kettunen@oulu.fi	FinnGen Analysis working group	FinnGen Analysis working group
Katri Pylkäs	University of Oulu, Oulu, Finland	katri.pylkas@oulu.fi	FinnGen Analysis working group	FinnGen Analysis working group
Eeva Sliz	University of Oulu, Oulu, Finland	eeva.sliz@oulu.fi	FinnGen Analysis working group	FinnGen Analysis working group
Minna Karjalainen	University of Oulu, Oulu, Finland	minna.k.karjalainen@oulu.fi	FinnGen Analysis working group	FinnGen Analysis working group
Tuomo Mantere	Northern Finland Biobank Borealis / University of Oulu / Northern Ostrobothnia Hospital District, Oulu, Finland	tuomo.mantere@oulu.fi	FinnGen Analysis working group	FinnGen Analysis working group
Eeva Kangasniemi	Finnish Clinical Biobank Tampere / University of Tampere / Pirkanmaa Hospital District, Tampere, Finland	eeva.kangasniemi@pshp.fi	FinnGen Analysis working group	FinnGen Analysis working group
Sami Heikkinen	University of Eastern Finland, Kuopio, Finland	sami.heikkinen@uef.fi	FinnGen Analysis working group	FinnGen Analysis working group
Arto Mannermaa	Biobank of Eastern Finland / University of Eastern Finland / Northern Savo Hospital District, Kuopio, Finland	arto.mannermaa@uef.fi	FinnGen Analysis working group	FinnGen Analysis working group
Eija Laakkonen	University of Jyväskylä, Jyväskylä, Finland	eija.k.laakkonen@jyu.fi	FinnGen Analysis working group	FinnGen Analysis working group
Nina Pitkänen	Auria Biobank / University of Turku / Hospital District of Southwest Finland, Turku, Finland	Niina.Pitkanen@tyks.fi	FinnGen Analysis working group	FinnGen Analysis working group
Samuel Lessard	Translational Sciences, Sanofi R&D, Framingham, MA, USA	samuel.lessard@sanofi.com	FinnGen Analysis working group	FinnGen Analysis working group
Clément Chatelain	Translational Sciences, Sanofi R&D, Framingham, MA, USA	clement.chatelain@sanofi.com	FinnGen Analysis working group	FinnGen Analysis working group
Perttu Terho	Auria Biobank / University of Turku / Hospital District of Southwest Finland, Turku, Finland	perttu.terho@tyks.fi	Biobank directors	Biobank directors
Sirpa Soini	THL Biobank / Finnish Institute for Health and Welfare (THL), Helsinki, Finland	sirpa.soini@thl.fi	Biobank directors	Biobank directors

Jukka Partanen	Finnish Red Cross Blood Service / Finnish Hematology Registry and Clinical Biobank, Helsinki, Finland	jukka.partanen@veripalvelu.fi	Biobank directors	Biobank directors
Eero Punkka	Helsinki Biobank / Helsinki University and Hospital District of Helsinki and Uusimaa, Helsinki	eero.punkka@hus.fi	Biobank directors	Biobank directors
Raisa Serpi	Northern Finland Biobank Borealis / University of Oulu / Northern Ostrobothnia Hospital District, Oulu, Finland	raisa.serpi@ppshp.fi	Biobank directors	Biobank directors
Sanna Siltanen	Finnish Clinical Biobank Tampere / University of Tampere / Pirkanmaa Hospital District, Tampere, Finland	sanna.siltanen@pshp.fi	Biobank directors	Biobank directors
Veli-Matti Kosma	Biobank of Eastern Finland / University of Eastern Finland / Northern Savo Hospital District, Kuopio, Finland	veli-matti.kosma@uef.fi	Biobank directors	Biobank directors
Teijo Kuopio	Central Finland Biobank / University of Jyväskylä / Central Finland Health Care District, Jyväskylä, Finland	teijo.kuopio@ksshp.fi	Biobank directors	Biobank directors
Anu Jalanko	Institute for Molecular Medicine Finland (FIMM), HiLIFE, University of Helsinki, Helsinki, Finland	anu.jalanko@helsinki.fi	FinnGen Teams	Administration
Huei-Yi Shen	Institute for Molecular Medicine Finland (FIMM), HiLIFE, University of Helsinki, Helsinki, Finland	huei-yi.shen@helsinki.fi	FinnGen Teams	Administration
Risto Kajanne	Institute for Molecular Medicine Finland (FIMM), HiLIFE, University of Helsinki, Helsinki, Finland	risto.kajanne@helsinki.fi	FinnGen Teams	Administration
Mervi Aavikko	Institute for Molecular Medicine Finland (FIMM), HiLIFE, University of Helsinki, Helsinki, Finland	mervi.aavikko@helsinki.fi	FinnGen Teams	Administration
Henna Palin	Finnish Clinical Biobank Tampere / University of Tampere / Pirkanmaa Hospital District, Tampere, Finland	henna.palin@pshp.fi	FinnGen Teams	Administration
Malla-Maria Linna	Helsinki Biobank / Helsinki University and Hospital District of Helsinki and Uusimaa, Helsinki	malla-maria.linna@hus.fi	FinnGen Teams	Administration
Mitja Kurki	Institute for Molecular Medicine Finland (FIMM), HiLIFE, University of Helsinki, Helsinki, Finland; Broad Institute, Cambridge, MA, United States	mkurki@broadinstitute.org	FinnGen Teams	Analysis
Juha Karjalainen	Institute for Molecular Medicine Finland (FIMM), HiLIFE, University of Helsinki, Helsinki, Finland	juha.karjalainen@helsinki.fi	FinnGen Teams	Analysis
Pietro Della Briotta Parolo	Institute for Molecular Medicine Finland (FIMM), HiLIFE, University of Helsinki, Helsinki, Finland	pietro.dellabriottaparolo@helsinki.fi	FinnGen Teams	Analysis
Arto Lehisto	Institute for Molecular Medicine Finland (FIMM), HiLIFE, University of Helsinki, Helsinki, Finland	arto.lehisto@helsinki.fi	FinnGen Teams	Analysis
Juha Mehtonen	Institute for Molecular Medicine Finland (FIMM), HiLIFE, University of Helsinki, Helsinki, Finland	juha.mehtonen@helsinki.fi	FinnGen Teams	Analysis
Wei Zhou	Broad Institute, Cambridge, MA, United States	wzhou@broadinstitute.org	FinnGen Teams	Analysis
Masahiro Kanai	Broad Institute, Cambridge, MA, United States	mkanai@broadinstitute.org	FinnGen Teams	Analysis
Mutaamba Maasha	Broad Institute, Cambridge, MA, United States	mmaasha@broadinstitute.org	FinnGen Teams	Analysis
Hannele Laivuori	Institute for Molecular Medicine Finland (FIMM), HiLIFE, University of Helsinki, Helsinki, Finland	hannele.laivuori@helsinki.fi	FinnGen Teams	Clinical Endpoint Development
Aki Havulinna	Institute for Molecular Medicine Finland (FIMM), HiLIFE, University of Helsinki, Helsinki, Finland; Finnish Institute for Health and Welfare (THL), Helsinki, Finland	aki.havulinna@helsinki.fi	FinnGen Teams	Clinical Endpoint Development
Susanna Lemmelä	Institute for Molecular Medicine Finland (FIMM), HiLIFE, University of Helsinki, Helsinki, Finland	susanna.lemmela@helsinki.fi	FinnGen Teams	Clinical Endpoint Development
Tuomo Kiiskinen	Institute for Molecular Medicine Finland (FIMM), HiLIFE, University of Helsinki, Helsinki, Finland	tuomo.kiiskinen@helsinki.fi	FinnGen Teams	Clinical Endpoint Development
L. Elisa Lahtela	Institute for Molecular Medicine Finland (FIMM), HiLIFE, University of Helsinki, Helsinki, Finland	laura.lahtela@helsinki.fi	FinnGen Teams	Clinical Endpoint Development
Mari Kaunisto	Institute for Molecular Medicine Finland (FIMM), HiLIFE, University of Helsinki, Helsinki, Finland	mari.kaunisto@helsinki.fi	FinnGen Teams	Communication
Elina Kilpeläinen	Institute for Molecular Medicine Finland (FIMM), HiLIFE, University of Helsinki, Helsinki, Finland	elina.kilpelainen@helsinki.fi	FinnGen Teams	E-Science
Timo P. Sipilä	Institute for Molecular Medicine Finland (FIMM), HiLIFE, University of Helsinki, Helsinki, Finland	timo.p.sipila@helsinki.fi	FinnGen Teams	E-Science
Oluwaseun Alexander Dada	Institute for Molecular Medicine Finland (FIMM), HiLIFE, University of Helsinki, Helsinki, Finland	alexander.dada@helsinki.fi	FinnGen Teams	E-Science
Awaisa Ghazal	Institute for Molecular Medicine Finland (FIMM), HiLIFE, University of Helsinki, Helsinki, Finland	awaisa.ghazal@helsinki.fi	FinnGen Teams	E-Science
Anastasia Kytölä	Institute for Molecular Medicine Finland (FIMM), HiLIFE, University of Helsinki, Helsinki, Finland	anastasia.shcherban@helsinki.fi	FinnGen Teams	E-Science
Rigbe Weldatsadik	Institute for Molecular Medicine Finland (FIMM), HiLIFE, University of Helsinki, Helsinki, Finland	rigbe.weldatsadik@helsinki.fi	FinnGen Teams	E-Science
Sanni Ruotsalainen	Institute for Molecular Medicine Finland (FIMM), HiLIFE, University of Helsinki, Helsinki, Finland	sanni.ruotsalainen@helsinki.fi	FinnGen Teams	E-Science
Kati Donner	Institute for Molecular Medicine Finland (FIMM), HiLIFE, University of Helsinki, Helsinki, Finland	kati.donner@helsinki.fi	FinnGen Teams	Genotyping
Timo P. Sipilä	Institute for Molecular Medicine Finland (FIMM), HiLIFE, University of Helsinki, Helsinki, Finland	timo.p.sipila@helsinki.fi	FinnGen Teams	Genotyping
Anu Loukola	Helsinki Biobank / Helsinki University and Hospital District of Helsinki and Uusimaa, Helsinki	anu.loukola@hus.fi	FinnGen Teams	Sample Collection Coordination
Päivi Laiho	THL Biobank / Finnish Institute for Health and Welfare (THL), Helsinki, Finland	paivi.laiho@thl.fi	FinnGen Teams	Sample Logistics
Tuuli Sistonen	THL Biobank / Finnish Institute for Health and Welfare (THL), Helsinki, Finland	tuuli.sistonen@thl.fi	FinnGen Teams	Sample Logistics
Essi Kaiharju	THL Biobank / Finnish Institute for Health and Welfare (THL), Helsinki, Finland	essi.kaiharju@thl.fi	FinnGen Teams	Sample Logistics
Markku Laukkanen	THL Biobank / Finnish Institute for Health and Welfare (THL), Helsinki, Finland	markku.laukkanen@thl.fi	FinnGen Teams	Sample Logistics
Elina Järvensivu	THL Biobank / Finnish Institute for Health and Welfare (THL), Helsinki, Finland	elina.jarvensivu@thl.fi	FinnGen Teams	Sample Logistics
Sini Lähteenmäki	THL Biobank / Finnish Institute for Health and Welfare (THL), Helsinki, Finland	sini.lahteenmaki@thl.fi	FinnGen Teams	Sample Logistics
Lotta Männikkö	THL Biobank / Finnish Institute for Health and Welfare (THL), Helsinki, Finland	lotta.mannikko@thl.fi	FinnGen Teams	Sample Logistics
Regis Wong	THL Biobank / Finnish Institute for Health and Welfare (THL), Helsinki, Finland	regis.wong@thl.fi	FinnGen Teams	Sample Logistics
Auli Toivola	THL Biobank / Finnish Institute for Health and Welfare (THL), Helsinki, Finland	auli.toivola@thl.fi	FinnGen Teams	Sample Logistics
Minna Brunfeldt	THL Biobank / Finnish Institute for Health and Welfare (THL), Helsinki, Finland	minna.brunfeldt@thl.fi	FinnGen Teams	Registry Data Operations
Hannele Mattsson	THL Biobank / Finnish Institute for Health and Welfare (THL), Helsinki, Finland	hannele.mattsson@thl.fi	FinnGen Teams	Registry Data Operations
Kati Kristiansson	THL Biobank / Finnish Institute for Health and Welfare (THL), Helsinki, Finland	kati.kristiansson@thl.fi	FinnGen Teams	Registry Data Operations
Susanna Lemmelä	Institute for Molecular Medicine Finland (FIMM), HiLIFE, University of Helsinki, Helsinki, Finland	susanna.lemmela@helsinki.fi	FinnGen Teams	Registry Data Operations
Sami Koskelainen	THL Biobank / Finnish Institute for Health and Welfare (THL), Helsinki, Finland	sami.koskelainen@thl.fi	FinnGen Teams	Registry Data Operations
Tero Hiekkalinna	THL Biobank / Finnish Institute for Health and Welfare (THL), Helsinki, Finland	tero.hiekkalinna@helsinki.fi	FinnGen Teams	Registry Data Operations
Teemu Paajanen	THL Biobank / Finnish Institute for Health and Welfare (THL), Helsinki, Finland	teemu.paajanen@thl.fi	FinnGen Teams	Registry Data Operations
Priit Palta	Institute for Molecular Medicine Finland (FIMM), HiLIFE, University of Helsinki, Helsinki, Finland	priit.palta@helsinki.fi	FinnGen Teams	Sequencing Informatics
Kalle Pärn	Institute for Molecular Medicine Finland (FIMM), HiLIFE, University of Helsinki, Helsinki, Finland	kalle.parn@helsinki.fi	FinnGen Teams	Sequencing Informatics
Mart Kals	Institute for Molecular Medicine Finland (FIMM), HiLIFE, University of Helsinki, Helsinki, Finland	mart.kals@helsinki.fi	FinnGen Teams	Sequencing Informatics
Shuang Luo	Institute for Molecular Medicine Finland (FIMM), HiLIFE, University of Helsinki, Helsinki, Finland	shuang.luo@helsinki.fi	FinnGen Teams	Sequencing Informatics
Tarja Laitinen	Pirkanmaa Hospital District, Tampere, Finland	tarja.laitinen@pshp.fi	FinnGen Teams	Trajectory
Mary Pat Reeve	Institute for Molecular Medicine Finland (FIMM), HiLIFE, University of Helsinki, Helsinki, Finland	mary.reeve@helsinki.fi	FinnGen Teams	Trajectory
Shanmukha Sampath Padmanabhuni	Institute for Molecular Medicine Finland (FIMM), HiLIFE, University of Helsinki, Helsinki, Finland	sam.padmanabhuni@helsinki.fi	FinnGen Teams	Trajectory
Marianna Niemi	University of Tampere, Tampere, Finland	marianna.niemi@tuni.fi	FinnGen Teams	Trajectory
Harri Siirtola	University of Tampere, Tampere, Finland	harri.siirtola@tuni.fi	FinnGen Teams	Trajectory

Javier Gracia-Tabuenca	University of Tampere, Tampere, Finland	javier.graciatabuenca@tuni.fi	FinnGen Teams	Trajectory
Mika Helminen	University of Tampere, Tampere, Finland	mika.helminen@tuni.fi	FinnGen Teams	Trajectory
Tiina Luukkaala	University of Tampere, Tampere, Finland	tiina.luukkaala@tuni.fi	FinnGen Teams	Trajectory
Iida Vähätalo	University of Tampere, Tampere, Finland	iida.vahatalo@epshp.fi	FinnGen Teams	Trajectory
Jyrki Pitkänen	Institute for Molecular Medicine Finland (FIMM), HiLIFE, University of Helsinki, Helsinki, Finland	jyrki.pitkanen@helsinki.fi	FinnGen Teams	Data protection officer
Marco Hautalahti	Finnish Biobank Cooperative - FINBB	marco.hautalahti@finbb.fi	FinnGen Teams	FINBB - Finnish biobank cooperative
Johanna Mäkelä	Finnish Biobank Cooperative - FINBB	johanna.makela@finbb.fi	FinnGen Teams	FINBB - Finnish biobank cooperative
Sarah Smith	Finnish Biobank Cooperative - FINBB	sarah.smith@finbb.fi	FinnGen Teams	FINBB - Finnish biobank cooperative
Tom Southerington	Finnish Biobank Cooperative - FINBB	tom.southerington@finbb.fi	FinnGen Teams	FINBB - Finnish biobank cooperative
