# Supplementary material for: Quantifying the causal impact of biological risk factors on healthcare costs
Source: Nat Commun. 2023 Sep 13;14:5672. doi: 10.1038/s41467-023-41394-4 (PMC10499912; doi:10.1038/s41467-023-41394-4)
Supplement: Supplementary file 3 — Reporting Summary [file 41467_2023_41394_MOESM3_ESM.pdf]

nature portfolio

Corresponding author(s): Andrea Ganna

Last updated by author(s): Jul 28, 2023

Reporting Summary

Nature Portfolio wishes to improve the reproducibility of the work that we publish. This form provides structure for consistency and transparency in reporting. For further information on Nature Portfolio policies, see our Editorial Policies and the Editorial Policy Checklist.

Statistics

For all statistical analyses, confirm that the following items are present in the figure legend, table legend, main text, or Methods section.

n/a	Confirmed	
[ ]	[x]	The exact sample size (n) for each experimental group/condition, given as a discrete number and unit of measurement
[x]	[ ]	A statement on whether measurements were taken from distinct samples or whether the same sample was measured repeatedly
[ ]	[x]	The statistical test(s) used AND whether they are one- or two-sided<br>*Only common tests should be described solely by name; describe more complex techniques in the Methods section.*
[ ]	[x]	A description of all covariates tested
[ ]	[x]	A description of any assumptions or corrections, such as tests of normality and adjustment for multiple comparisons
[ ]	[x]	A full description of the statistical parameters including central tendency (e.g. means) or other basic estimates (e.g. regression coefficient) AND variation (e.g. standard deviation) or associated estimates of uncertainty (e.g. confidence intervals)
[ ]	[x]	For null hypothesis testing, the test statistic (e.g. F, t, r) with confidence intervals, effect sizes, degrees of freedom and P value noted<br>*Give P values as exact values whenever suitable.*
[x]	[ ]	For Bayesian analysis, information on the choice of priors and Markov chain Monte Carlo settings
[x]	[ ]	For hierarchical and complex designs, identification of the appropriate level for tests and full reporting of outcomes
[x]	[ ]	Estimates of effect sizes (e.g. Cohen's d, Pearson's r), indicating how they were calculated

Our web collection on statistics for biologists contains articles on many of the points above.

Software and code

Policy information about availability of computer code

Data collection	No open source or custom code was used to collect the data in this study.
Data analysis	All code used to collect the data is available via Github: https://github.com/jiwooleebroadinstitute/healthcare_cost_finngen (DOI 10.5281/zenodo.8184260). Genome-wide association studies were performed using REGENIE version v.3.2.8. Mendelian Randomization was performed using the TwoSampleMR package version 0.5.6 in R version 4.1. Genetic correlation was calculated using command-line tool, LDSC version 1.0.0. Data visualization was performed using ggplot package version 3.4.2 in R version 4.1.

For manuscripts utilizing custom algorithms or software that are central to the research but not yet described in published literature, software must be made available to editors and reviewers. We strongly encourage code deposition in a community repository (e.g. GitHub). See the Nature Portfolio guidelines for submitting code & software for further information.

Data

Policy information about availability of data

All manuscripts must include a data availability statement. This statement should provide the following information, where applicable:

- Accession codes, unique identifiers, or web links for publicly available datasets
- A description of any restrictions on data availability
- For clinical datasets or third party data, please ensure that the statement adheres to our policy

All data are made available from the FinnGen Study with research proposals approved by institutional review board and the FinnGen Scientific Committee. Participants in FinnGen provided informed consent for biobank research on basis of the Finnish Biobank Act. The Coordinating Ethics Committee of the Hospital

District of Helsinki and Uusimaa (HUS) approved the FinnGen study protocol (number HUS/990/2017). The FinnGen study is approved by the THL (approval number THL/2031/6.02.00/2017, amendments THL/1101/5.05.00/2017, THL/341/6.02.00/2018, THL/2222/6.02.00/2018, THL/283/6.02.00/2019 and THL/1721/5.05.00/2019), the Digital and Population Data Service Agency (VRK43431/2017-3, VRK/6909/2018-3 and VRK/4415/2019-3), the Social Insurance Institution (KELA) (KELA 58/522/2017, KELA 131/522/2018, KELA 70/522/2019 and KELA 98/522/2019) and Statistics Finland (TK-53-1041-17). Supporting genome-wide association data supporting the findings of this study are publicly available on request from the UK Biobank (https://www.ukbiobank.ac.uk/) and the Netherlands Twin Register (https://www.nimhgenetics.org/download-tool/NTR). All source data are provided with this paper.

Research involving human participants, their data, or biological material

Policy information about studies with human participants or human data. See also policy information about sex, gender (identity/presentation), and sexual orientation and race, ethnicity and racism.

Reporting on sex and gender	This study was compliant with the policy on reporting on sex and gender. Sex was self-reported and carefully considered in our study and the statistical models include sex as a covariate adjustment used to estimate the association between genetic variants and healthcare costs as well as the causal effects between exposures and healthcare costs. We reported causal estimates stratified by sex in Figure 4B.
Reporting on race, ethnicity, or other socially relevant groupings	This study was compliant with the policy in reporting race, ethnicity, or other socially relevant groupings. Ancestry was considered using principal components of genetic ancestry and the statistical models included ancestry as a covariate adjustment in the genome-wide association analyses. This study did not analyze differences between socially constructed groupings and did not use them as proxies for other features.
Population characteristics	We included 373,160 FinnGen participants (data freeze 8) followed-up to a maximum of 22 years. The average age at baseline (i.e., date of DNA sample collection) was 54 years old and 56% of the study cohort was female.
Recruitment	This study utilized data from the FinnGen Study, which is an ongoing prospective cohort study aiming to recruit 520,000 individuals by combining population-based legacy cohorts, disease-based cohorts, and volunteers recruited by biobanks. The average age at baseline (i.e., date of DNA sample collection) is 54 years old and 56% of the study cohort is female. Participants are linked to national health registries that provide rich longitudinal information. Such registries include the Register of Primary Health Care Visits (AvoHILMO) which captures outpatient visits, the Care Register for Health Care (HILMO) which captures hospital visits, and the Medication Reimbursement Register (Kela). Individual-level genotypes and register data from FinnGen participants can be accessed by approved researchers via the Fin-genious portal (https://site.fingenious.fi/en/) hosted by the Finnish Biobank Cooperative FinBB (https://finbb.fi/en/). Data release to FinBB is timed to the bi-annual public release of FG summary results which occurs twelve months after FG consortium members can start working with the data.<br><br>Given that the study participants in FinnGen may differ from the entire Finnish population due to its hospital-based recruitment (e.g., individuals in FinnGen are typically sicker and have higher disease prevalence), we adjusted the study cohort in FinnGen to the entire Finnish population using inverse probability weights in a subsequent sensitivity analysis. We used the calibration weighting method, which uses the marginal proportions of variables to adjust the sample weights to satisfy the population margins. We used the following five health and sociodemographic characteristics: age, gender, education, occupation, and region of birth.
Ethics oversight	The Coordinating Ethics Committee of the Hospital District of Helsinki and Uusimaa (HUS) approved the FinnGen study protocol (number HUS/990/2017). The FinnGen study is approved by the THL (approval number THL/2031/6.02.00/2017, amendments THL/1101/5.05.00/2017, THL/341/6.02.00/2018, THL/2222/6.02.00/2018, THL/283/6.02.00/2019 and THL/1721/5.05.00/2019), the Digital and Population Data Service Agency (VRK43431/2017-3, VRK/6909/2018-3 and VRK/4415/2019-3), the Social Insurance Institution (KELA) (KELA 58/522/2017, KELA 131/522/2018, KELA 70/522/2019 and KELA 98/522/2019) and Statistics Finland (TK-53-1041-17).

Note that full information on the approval of the study protocol must also be provided in the manuscript.

Field-specific reporting

Please select the one below that is the best fit for your research. If you are not sure, read the appropriate sections before making your selection.

☒ Life sciences ☐ Behavioural & social sciences ☐ Ecological, evolutionary & environmental sciences

For a reference copy of the document with all sections, see nature.com/documents/nr-reporting-summary-flat.pdf

Life sciences study design

All studies must disclose on these points even when the disclosure is negative.

Sample size	In FinnGen, 373,160 individuals after data exclusion were used. In the UK Biobank, 307,048 individuals after data exclusion were used. In the Netherlands Twin Register, 16,726 individuals were used after data exclusion. Sample sizes were determined by the number of individuals who were genotyped and passed quality control. The number of individuals used sufficient statistical power to detect significant causal effects between exposures and outcomes.
Data exclusions	Individuals with end of follow-up before baseline, without genetic data, and without cost data were excluded.
Replication	Replication studies were successfully conducted both in the UK Biobank and Netherlands Twin Register. Using the available-upon-request

	summary statistics from the UK Biobank and Netherlands Twin Register, the Mendelian Randomization pipeline used in FinnGen was used to calculate causal estimates of waist circumference, body mass index, and systolic blood pressure on annual total healthcare costs using the exact same methods.
Randomization	Not applicable. This is an observational study.
Blinding	Not applicable. This is an observational study.

Reporting for specific materials, systems and methods

We require information from authors about some types of materials, experimental systems and methods used in many studies. Here, indicate whether each material, system or method listed is relevant to your study. If you are not sure if a list item applies to your research, read the appropriate section before selecting a response.

Materials & experimental systems

n/a	Involved in the study	
☒	☐	Antibodies
☒	☐	Eukaryotic cell lines
☒	☐	Palaeontology and archaeology
☒	☐	Animals and other organisms
☒	☐	Clinical data
☒	☐	Dual use research of concern
☒	☐	Plants

Methods

n/a	Involved in the study	
☒	☐	ChIP-seq
☒	☐	Flow cytometry
☒	☐	MRI-based neuroimaging
